# Functional mapping of the 14-3-3 hub protein as a guide to design 14-3-3 molecular glues†

**DOI:** 10.1039/d2sc04662h

**Published:** 2022-10-25

**Authors:** Bente A. Somsen, Fenna W. B. Craenmehr, Wei-Hong W. Liu, Auke A. Koops, Marloes A. M. Pennings, Emira J. Visser, Christian Ottmann, Peter J. Cossar, Luc Brunsveld

**Affiliations:** Laboratory of Chemical Biology, Department of Biomedical Engineering and Institute for Complex Molecular Systems, Eindhoven University of Technology P.O. Box 513 Eindhoven 5600 MB The Netherlands p.cossar@tue.nl l.brunsveld@tue.nl

## Abstract

Molecular glues represent an evolution in drug discovery, however, targeted stabilization of protein complexes remains challenging, owing to a paucity of drug design rules. The functional mapping of hotspots has been critical to protein–protein interaction (PPI) inhibitor research, however, the orthogonal approach to stabilize PPIs has not exploited this information. Utilizing the hub protein 14-3-3 as a case study we demonstrate that functional mapping of hotspots provides a triage map for 14-3-3 molecular glue development. Truncation and mutation studies allowed deconvoluting the energetic contributions of sidechain and backbone interactions of a 14-3-3-binding non-natural peptide. Three central 14-3-3 hotspots were identified and their thermodynamic characteristics profiled. In addition to the phospho-binding pocket; (i) Asn226, (ii) Lys122 and (iii) the hydrophobic patch formed by Leu218, Ile219 and Leu222 were critical for protein complex formation. Exploiting this hotspot information allowed a peptide-based molecular glue that elicits high cooperativity (*α* = 36) and selectively stabilizes the 14-3-3/ChREBP PPI to be uniquely developed.

## Abstract 2

The functional mapping of a 14-3-3 protein complex, by means of peptide truncations and point mutations, as an approach to identify critical hotspots regions for 14-3-3 molecular glue drug design.

## Graphical abstract

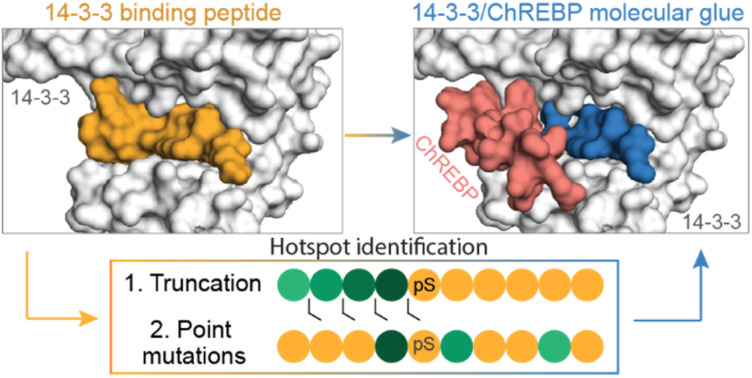

## Introduction

Molecular glues represent a breakthrough in drug discovery, enabling non-disruptive control of protein function. The core advancement is the small-molecule strengthening of native protein complexes or induction of non-native protein–protein interactions (PPIs). Stabilization of a protein complex typically results in enhanced cellular or a new cellular function.^1–3^ This new drug modality expands the drug discovery chemical toolbox and enables previously undruggable protein targets to be addressed. Molecular glues were first elucidated from mode-of-action studies of naturally derived macrocycles and tetracyclic compounds; including rapamycin,^4,5^ FK506,^6^ cyclosporin^6–8^ and taxol.^9,10^

Medicinal chemistry campaigns have addressed non-native protein complexes, most notably molecular glue degraders such as immunomodulatory drugs (IMiDs) and aryl sulfonylamides, for example targeting cereblon-CUL4 and Cullin ring E3 ligases.^11–15^ The stabilization of native protein–protein interactions has simultaneously gained significant attention, for example with the development of NRX-103094 and Trametiglue that stabilize mutant β-catenin/β-TrCP and KSR/MEK protein complexes, respectively.^16,17^ Further, stabilization of hub protein 14-3-3 interactions with the p65 domain of NF-κB, chloride channel CFTR, and ChREBP transcription factor have also been demonstrated as promising approaches to molecular glue drug discovery.^18–20^

These forerunners in the field stand as valuable proofs of concept to the emerging molecular glue drug discovery field. However, systematic drug discovery approaches to target native protein complexes remain to be developed. Three challenges complicate initial molecular glue chemical matter identification: (1) a lack of native ligands and tool compounds, (2) seemingly featureless binding interfaces and (3) the dynamic nature of multi-component protein complexes. To address these challenges a greater thermodynamic understanding of protein complex formation is necessary.

**Fig. 1:**
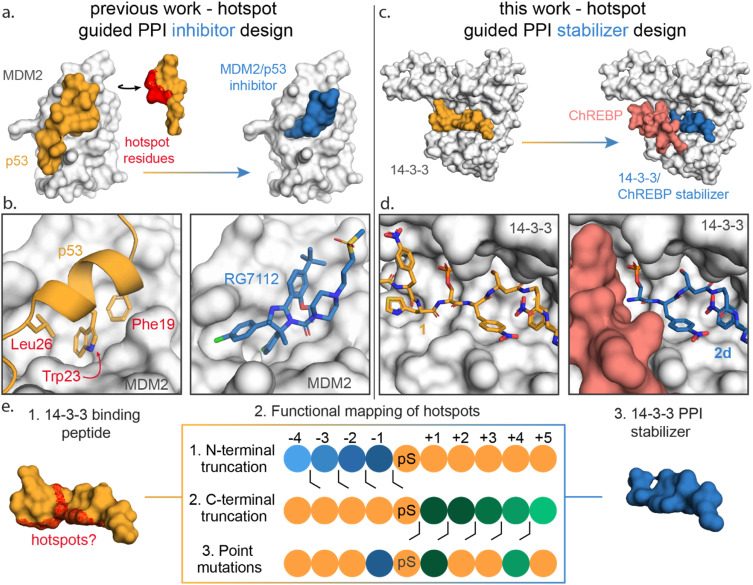
(a) Conceptual representation of PPI inhibitor design based on hotspot residue analysis, using the MDM2/p53 PPI as an example.^29,31^ Hotspot residues (red region) for MDM2 (white surface) binding were determined for the p53 protein (orange). Based on these hotspots small molecule inhibitors such as RG7112 (blue) were designed to inhibit the MDM2/p53 interaction. (b) Enlarged and detailed view on MDM2 (white surface)/p53 (orange cartoon) interaction with hotspot residues Phe19, Trp23 and Leu26 and the MDM2/RG7112 (blue sticks) interaction is depicted (PDB: 1YCR & 4IPF). (c and d) Conceptual representation of this work where a PPI stabilizer is designed based on hotspot residue analysis. Hotspot residues for 14-3-3 (white surface) binding were determined for peptide 1 (orange). Based on these hotspots a peptide-based stabilizer (2d, blue) was designed for the 14-3-3/ChREBP PPI (PDB: 6TCH, 7ZMU & 5F74). (e) Schematic representation of the functional mapping of hotspots, based on truncation studies and point mutations, to determine hotspot residues in 14-3-3 binding peptide, for the design of a 14-3-3 PPI stabilizer.

Functional mapping of protein interfaces has proven invaluable to the mechanistic understanding of protein complex formation and PPI inhibition drug discovery (Fig. 1a).^21–23^ Alanine scanning mutagenesis, for example, shows that not all PPI interfacial amino acids are uniform in their energetic contribution; discrete regions, termed hotspots, disproportionately contribute to the binding energy of protein complex formation.^24–26^ Hotspot-based drug design has been applied to PPI inhibition, *e.g.* for the MDM2/p53 interaction to mimic hotspot residues Phe19, Trp23 and Leu26 of p53 using both peptide stapling^27^ and small molecules such as the nutlin member RG7112 (Fig. 1b).^28–32^ Further, hotspot-based drug design was used to develop von Hippel–Lindau (VHL) targeting ligands, significant to PROTAC research. There the 3-hydroxyl-l-proline (Hyp) 564 of hypoxia-inducible factor 1*α* was mimicked.^33,34^ Surprisingly, given the success of hotspot analysis for PPI inhibitor development, this concept has not received strong attention for molecular glue drug design.

Previously we have identified initial chemical starting points for 14-3-3 molecular glue design using covalent tethering.^18,35–37^ However optimization of molecular glues in the absence of well-defined hotspot residues is time and labor intensive. Functional mapping of the 14-3-3 binding groove would enable efficient fragment optimization to lead compounds.

Here we disclose how functional mapping of a 14-3-3 protein complex can be used to rapidly develop a peptide-based molecular glue for the 14-3-3/ChREBP protein complex (Fig. 1c and d). Hotspot analysis, by means of truncation and mutation studies, of 14-3-3 in complex with a binding peptide enabled critical interfacial residues to be prioritized in developing a non-natural peptide stabilizer of the 14-3-3/ChREBP protein complex (Fig. 1e). Further, functional mapping and thermodynamic analysis provided mechanistic insight into 14-3-3 binding of disordered regions of phosphoproteins.

## Results and discussion

The hub protein 14-3-3 binds hundreds of different protein partners, typically in phosphorylated and structurally disordered regions of its client proteins.^38^ Because of its role in regulating many protein partners, 14-3-3 PPIs have become attractive drug discovery targets for various diseases including cancer and neurodegenerative diseases.^39,40^ Functional mapping of the 14-3-3 binding groove would therefore be valuable in both the design of PPI modulators and in gaining a better understanding of 14-3-3 PPI formation.

To functionally map the 14-3-3 binding interface, we sought a high affinity 14-3-3 binding peptide that would allow accurate *K*_D_ value determinations despite affinity losses during point mutation and truncation studies. Previously, Quartararo *et al.* had identified highly potent non-canonical phosphopeptides with low nanomolar affinity (3–19 nM) from a 10^8^-membered synthetic peptide library. Each library member contained a fixed phosphoserine, flanked by four variable non-canonical amino acids on each side. A C-terminal lysine was added to improve the sequencing results of hit peptides (ESI Fig. S1†).^41^

**Fig. 2:**
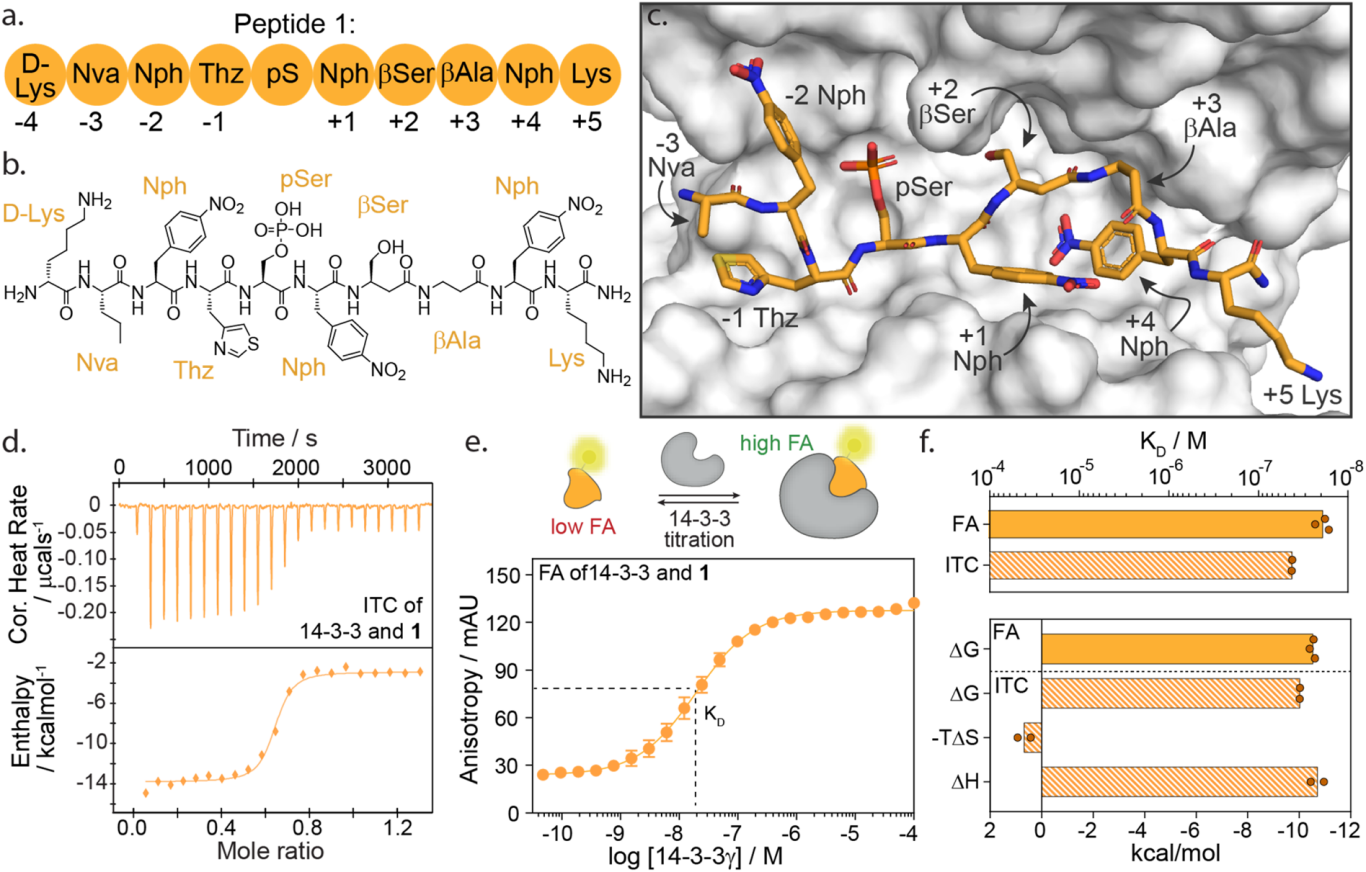
(a) Schematic representation of peptide 1 with the naming of individual amino acids and the according numbering system. Lys = lysine; Nva = norvaline; Nph = nitrophenylalanine; Thz = thiazolylalanine; pS = phosphoserine; βSer = beta-serine; βAla = beta-alanine. (b) Chemical structure of peptide 1. (c) Crystal structure of 14-3-3σ (white surface) bound to peptide 1 (orange sticks) (PDB: 6TCH). (d) Isothermal titration calorimetry (ITC) raw thermogram of 100 μM 14-3-3γ binding to 20 μM 1. (e) Fluorescence anisotropy (FA) binding curve of 14-3-3γ titration to fluorescein-labelled 1 (10 nM), including a schematic representation of FA concept. (f) Bar plot representation of binding affinity (*K*_D_) and thermodynamic parameters (Δ*G*, −*T*Δ*S*, Δ*H*) parameters as obtained from ITC and FA experiments.

We selected peptide 14-3-3.12, herein termed peptide 1, from the top hits for functional mapping. Peptide 1 was selected based on its high affinity (19 nM) and known X-ray crystal structural characterization data in complex with 14-3-3 (Fig. 2a–c). Further, the peptide completely occupies the 14-3-3 binding groove, thus allowing to explore both sides flanking 14-3-3's central phospho-accommodating pocket.

### High-affinity binding of peptide 1 is enthalpically driven

First, we sought to understand the thermodynamic properties of the high affinity 14-3-3/1 interaction. To analyze this interaction, we performed both isothermal titration calorimetry (ITC) and fluorescence anisotropy (FA) assays (Fig. 2d–f). FA and ITC were in close correlation with each other affording a *K*_D_ value of 19.2 ± 4 nM and 45.0 ± 1 nM, respectively (Fig. 2f and ESI Table S1†). These results correspond to free binding energy values Δ*G*, of −10.5 and −10.0 kcal mol^−1^ for the 14-3-3/1 interaction using FA and ITC, respectively. Interestingly, deconvolution of the Δ*G* into the thermodynamic parameters Δ*H* and Δ*S*, showed that the total Gibbs free energy is entirely driven by an enthalpic contribution, with a minor entropic penalty paid for peptide binding (Fig. 2f).

### The central role of the phosphate-binding pocket

In most 14-3-3 PPIs, the client proteins bind *via* a phosphorylated serine or threonine residue located in a disordered region of the client protein, for example in unstructured protein domains of the cystic fibrosis transmembrane conductance regulator (CFTR),^42^ the death-associated protein kinase 2 (DAPK2)^43^ or the neural precursor cell expressed developmentally down-regulated 4 ligase (Nedd4-2).^44^ The phosphorylated amino acid binds a small sub-pocket, formed by Arg56, Arg129 and Tyr130, within the amphipathic groove of 14-3-3 (ESI Fig. S2a†). To investigate the contribution of the phosphate group to 14-3-3 binding, we synthesized 1, where the phosphate group was removed (Ser). Further, we investigated the replacement of the phosphoserine with negatively charged glutamic acid (Glu) and aspartic acid (Asp) mutants. Aspartic acid has previously been reported as a potential phosphomimetic for 14-3-3 binding.^45^ All peptide modifications led to a substantial loss of 14-3-3 binding (ESI Fig. S2b†). This strongly suggests that the electrostatic interaction is essential for relevant 14-3-3 binding by such flexible phospho-motifs. The phosphate interaction appears to function as the nucleation site for the subsequent structural arrangement of the flanking peptide regions within the amphipathic groove of 14-3-3.

### Elucidation of crucial C- and N-terminal epitopes for 14-3-3 binding

**Fig. 3:**
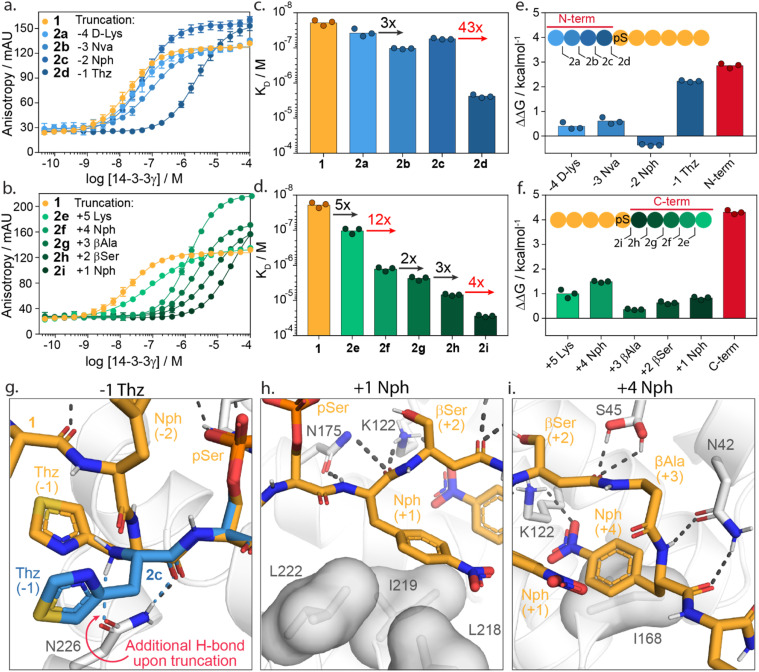
Truncation studies. (a) Fluorescence anisotropy assay of peptide 1 (orange) and N-terminally truncated peptides 2a–d (blue) in which 14-3-3γ is titrated to fixed concentrations fluorescein-labelled peptide (10 nM). (b) Fluorescence anisotropy assay of peptide 1 (orange) and C-terminally truncated peptides 2e–i (green) in which 14-3-3γ is titrated to fixed concentrations of fluorescein-labelled peptide (10 nM). (c and d) Bar plot representation from obtained binding affinities (*K*_D_) of truncated peptides and the fold change in affinity between different constructs. Data shows *K*_D_ values obtained from three independent experiments (*n* = 3). (e and f) Energy contribution analysis of individual amino acids of peptide 1 (blue/green) and the overall N- and C-terminal (red), based on the binding affinities observed in (a–d) and represented as ΔΔ*G*. A positive value indicates enhanced 14-3-3 binding whereas a negative value represents a reduction in 14-3-3 binding affinity. (g) Crystal structure analysis of amino acid Thz(−1) of 1 (orange sticks) and Thz(−1) of truncated peptide 2c (blue sticks) bound to 14-3-3σ (white cartoons and sticks). Favorable hydrogen bond and electrostatic interactions are shown with black or blue dashed lines (PDB: 6THC & 7ZMW). (h and i) Crystal structure analysis of amino acids Nph(+1) and Nph(+4) of the 1 (orange sticks) bound to 14-3-3 (white cartoon, surface and sticks). Favorable hydrogen bond and electrostatic interactions are shown with black dashed lines (PDB: 6TCH).

To investigate the energy contributions of amino acid residues flanking the phosphoserine of 1 truncation studies of the C- and N-termini were performed (Fig. 3a–f). Truncation from the N-terminus provided four peptides 2a–d (ESI Fig. S3†) and from the C-terminus five peptides 2e–i (ESI Fig. S4†). Affinities for 14-3-3 of these peptides were evaluated using a FA assay (Fig. 3a–d, ESI Tables S2 and S3†). Based on the determined *K*_D_ values of each truncated peptide the individual energy contribution of each residue in peptide 1 was determined (Fig. 3e and f). The individual energy contribution for each amino acid was then calculated by subtracting the Δ*G* values (based on the *K*_D_) of the truncated peptide from the previously truncated peptide, providing the ΔΔ*G*. Furthermore, the cumulative energy contributions of all N- or C-terminal amino acids were also calculated. This provided an overall ΔΔ*G* of the N- and C-terminal amino acids respective to the 9-mer phosphorylated peptide 1. N-terminal amino acids d-Lys(−4, 2a), Nva(−3, 2b, Norvaline) and Nph(−2, 2c, 4-nitrophenylanline) showed only minor energy contributions to 14-3-3 binding. In contrast, truncations of Thz (−1, 2d, dl-4-thiazolylalanine) elicited a 43-fold change in binding affinity (Fig. 3c). This fold change translates to an individual ΔΔ*G* of 2.2 kcal mol^−1^, the greatest binding energy contribution of the N-terminal amino acids (Fig. 3e). Moreover, except pSer, Thz(−1) is the most significant contributor to the overall affinity of 1 when comparing individual energy contributions across the whole peptide. Truncation of the entire N-terminus resulted in a ΔΔ*G* of 2.9 kcal mol^−1^ (Fig. 3e).

Truncation of the C-terminal amino Lys(+5, 2e) gave a five-fold reduction in binding affinity compared to 1. Removal of Nph(+4, 2f) resulted in an even stronger change in affinity (12-fold, ΔΔ*G* = 1.5 kcal mol^−1^). The β-Ala(+3, 2g) and β-Ser(+2, 2h) residues contributed the least, with ΔΔ*G* = 0.4 kcal mol^−1^ and 0.6 kcal mol^−1^, respectively. Nph(+1, 2i) gave a slightly more significant fold change in binding affinity (four-fold) and proved to be more important for 14-3-3 binding. The entire C-terminus affords a cumulative ΔΔ*G* of 4.3 kcal mol^−1^. Interestingly, this presents an opportunity for 14-3-3 molecular glue design as many native 14-3-3 client proteins are either C-terminally truncated or feature a C-terminus that bends out of the 14-3-3 binding groove (ESI Fig. S5†). Given that most molecular glues identified so far occupy this empty section of the 14-3-3 groove, they sit in proximity to these critical hotspots. Notably, the higher energy contribution of the C-terminus compared with the N-terminal part of 1 is in contrast with native 14-3-3 binders. We speculate this is a constraint of peptides 1 synthetic design; potentially higher affinity peptide binders would exploit additional hotspots with the N-terminal section. However, such peptide starting points would be less interesting to 14-3-3 molecular glue design.

Structural analysis of the X-ray crystal structure of 1 with 14-3-3σ provides insight into the molecular interactions of the Thz(−1), Nph(+1) and Nph(+4), which showed to most significantly contribute to 14-3-3 binding (Fig. 3g–i). The backbone carbonyl of Thz(−1) forms a hydrogen bond with the side chain of Asn226 of 14-3-3 (Fig. 3g). Truncating peptide 1 at Thz(−1) (peptide 2c) resulted in a conformational change of Thz(−1), creating an additional hydrogen bond between the N-terminus of Thz(−1) and Asn226. We hypothesize that the alignment of the −1 amino acid backbone to Asn226 of 14-3-3 leads to the strong contribution of this −1 amino acid to 14-3-3 binding. The further truncation of the peptide results in a loss of both hydrogen bonds (peptide 2d, ESI Fig. S6†). Analysis of the Nph(+1) molecular interactions shows that the residue makes hydrophobic interactions with the amino acids Leu218, Ile219 and Leu222 of 14-3-3. Additionally, the backbone atoms of Nph(+1) form hydrogen bonds with Asn175 and Lys122 (Fig. 3h). Analysis of Nph(+4) shows that this residue occupies a sub-pocket of the amphipathic groove of 14-3-3 shaped by residues Phe119, Lys122, Ile168 and Gly171. Notably, the nitro functionality forms an electrostatic interaction with Lys122 (Fig. 3i). This result is in line with earlier identified PPI stabilizers, such as the pyrrolidones, imine- and disulfide tethering fragments, that bind within this sub-pocket and engage with Lys122 (ESI Fig. S7†).^18,35–37,46^

### Localizing 14-3-3 hotspot residues at the protein–protein interface

**Fig. 4:**
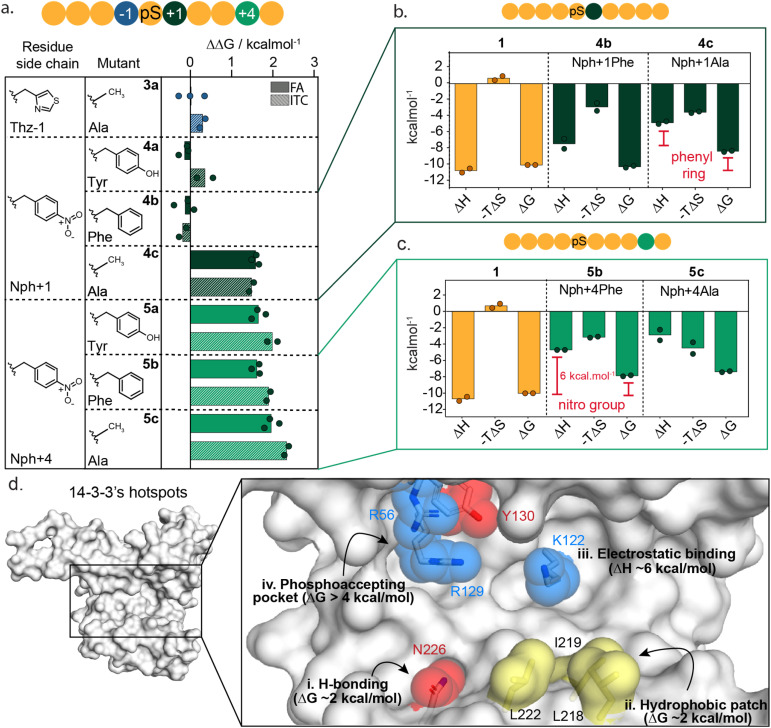
Mutational studies around Thz(−1), Nph(+1) and Nph(+4) residues. (a) Energy contribution analysis of point mutated Thz(−1) (3a, blue), Nph+1 (4a–c, dark green) and Nph+4 (5a–c, light green) residues in 1 to tyrosine, phenylalanine and alanine residues, represented as ΔΔ*G*. Data represents both results from fluorescence anisotropy (FA) and isothermal titration calorimetry (ITC). (b) Thermodynamic parameters (Δ*H*, −*T*Δ*S* and Δ*G* in kcal mol^−1^) determined by ITC assays for peptide 1 and Nph+1 mutants 4b,c. Thermodynamic parameters are reported from two independent experiments (*n* = 2). (c) Thermodynamic parameters (Δ*H*, −*T*Δ*S* and Δ*G* in kcal mol^−1^) determined by ITC assays for peptide 1 and Nph+1 mutants 5b-c. Thermodynamic parameters are reported from two independent experiments (*n* = 2). (d) Hotspot analysis map of 14-3-3σ binding groove showing most relevant residues for the interaction with 14-3-3 binding partners. Red amino acids form hydrogen bonds with a binding partner, blue residues facilitate electrostatic interactions and yellow amino acids represent residues that form hydrophobic contacts.

To further dissect the specific functional group interactions of Thz(−1), Nph(+1) and Nph(+4) a point mutation study was performed (Fig. 4a and ESI Fig. S8†). Specifically, Thz(−1) was mutated to an alanine (peptide 3a) to investigate the role of the thiazolyl side chain in peptide binding. Nitrophenylalanine residues Nph(+1) and Nph(+4) were each mutated to tyrosine (Tyr), phenylalanine (Phe) and alanine (Ala) to deconvolute electrostatic, hydrogen bond and hydrophobic interactions (peptides 4a–c and 5a–c). Binding studies of the resulting mutated peptides were performed using FA assays and ITC experiments (ESI Fig. S9 and S10†). The derived *K*_D_ values (ESI Tables S4 and S5†), were used to calculate the individual energy contributions (ΔΔ*G*) of the side group functionalities (Fig. 4a and ESI Fig. S11†).

Comparison of *K*_D_ values from FA and ITC assays aligned well for all mutants (Fig. 4a). The removal of the thiazolyl ring of Thz(−1) residue (3a) had no impact on 14-3-3 binding affinity, with ΔΔ*G* 0.0 ± 0.4 kcal mol^−1^ (FA, Fig. 4a, blue) which contrasts significantly with the ΔΔ*G* of 2.2 kcal mol^−1^ observed upon complete removal of Thz(−1) (Fig. 3e). This result indicates that most binding affinity at this position is contributed by the backbone heavy atoms in the −1 amino acid, and that the side chain does not contribute to affinity. Crystallography studies support this observation with Thz(−1) backbone engaging in hydrogen bonding with Asn226 of 14-3-3 (Fig. 3g). This identifies Asn226 as crucial hotspot within the 14-3-3 binding groove (Fig. 4d).

Unsurprisingly, given the solvent exposure of the nitro group (Fig. 3h), substituting Nph(+1) to Phe or Tyr (4a,b) showed no significant change in affinity (Fig. 4a, dark green) with ΔΔ*G* values of −0.1 ± 0.3 and −0.2 ± 0.2 kcal mol^−1^ (FA experiments), respectively. The slightly negative values even indicate a minor improvement in the binding affinity of this mutant peptide. Analysis of the thermodynamic parameters showed that removal of the nitro group led to an enhanced entropic contribution relative to 1 (Fig. 4b and ESI Table S6†). We hypothesize the negative charge Nph (+1) is not complementary to the hydrophobic patch and the removal of the nitro group eliminates this entropic penalty. In contrast to the removal of the nitro-group, removing the complete phenyl ring (4c) showed a significant loss in affinity, with ΔΔ*G* of 1.8 ± 0.1 and 1.7 ± 0.1 kcal mol^−1^ for FA and ITC, respectively. Thermodynamic analysis of the alanine mutant (4c) relative to the phenylalanine mutant (4b) showed the phenyl ring purely contributes to the enthalpy of binding, whereas the entropy did not change significantly (Fig. 4b and ESI Table S6†). These results illustrate that the hydrophobic patch, shaped by L218, I219 and L222 of 14-3-3 are critical residues to target when one wants to increase the affinity of small molecule modulators of 14-3-3 PPIs (Fig. 4d(ii)).

In contrast to Nph(+1), removing the nitro group of the +4 residue (5b) significantly decreased the binding affinity to 14-3-3 (Fig. 4a and ESI Fig. S9–S11†); resulting in a ΔΔ*G* 2.3 ± 0.2 kcal mol^−1^ in ITC studies. Replacement of the nitro for the hydroxy group (5a, Nph(+4) Tyr) did not restore the binding affinity. Comparing the thermodynamic parameters of peptide 1 (nitrophenylalanine) to 5b (phenylalanine) shows that the removal of the nitro group significantly increases the enthalpy by ∼6 kcal mol^−1^ (Fig. 4c), albeit with an accompanying partial enthalpy-entropy compensation. These observations indicate that the enthalpy contribution of the electrostatic interaction between the nitro group of Nph(+4) and Lys122 of 14-3-3 (Fig. 3i) dominates this subpocket binding interaction, making Lys122 a clear hotspot in 14-3-3 (Fig. 4d(iii)).

### A cooperative peptide molecular glue for the 14-3-3/ChREBP PPI

Having identified hotspot regions within the 14-3-3 binding groove, we envisioned this information could be used to identify and optimize 14-3-3/ChREBP molecular glues. The binding of 14-3-3 to ChREBP (Carbohydrate-Response Element Binding Protein) has shown to inhibit nuclear localization of ChREBP thereby decreasing its transcriptional activity of glucose responsive genes.^47^ Previous studies have shown that stabilization of the 14-3-3/ChREBP protein complex, using the natural metabolite AMP or synthetic stabilizers, is valuable to regulate ChREBP binding or activity.^20,47^

**Fig. 5:**
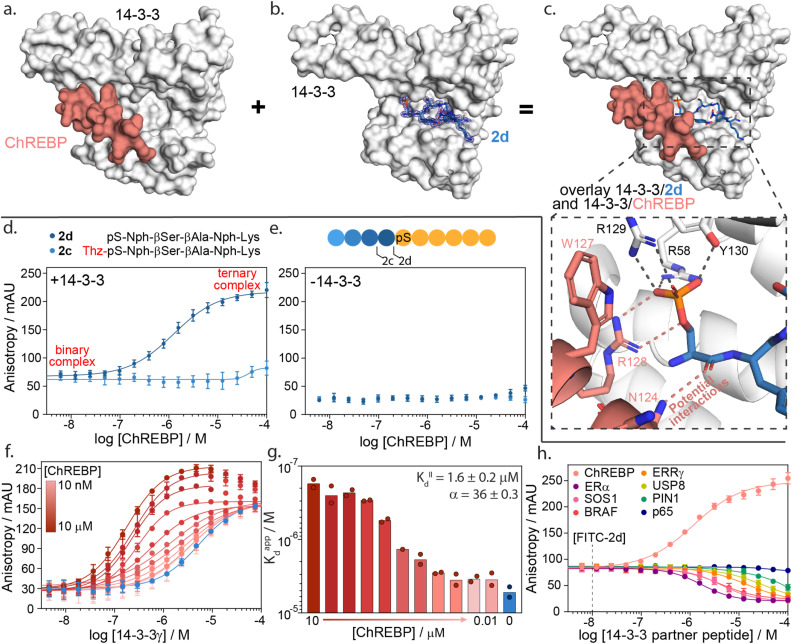
14-3-3γ/ChREBP PPI stabilization by non-natural truncated peptide 2d. (a) Crystal structure of 14-3-3 (white surface) bound to ChREBP peptide (salmon sticks and cartoon), (PDB: 5F74). (b) Crystal structure of 14-3-3 (white surface) bound to 2d (blue sticks). The final 2Fo-Fc electron density map is represented as a blue mesh contoured at 1*σ* (PDB: 7ZMU). (c) Structural overlay of 14-3-3 bound to 2d and ChREBP showing a strong binding complementary in the 14-3-3 binding groove. An enlarged view is given of the phosphate group of peptide 2d interacting with the phospho-accepting pocket of 14-3-3 (black dashes) and potential interactions between 2d and ChREBP (salmon dashes) (PDB 7ZMU & 5F74). (d) Fluorescence anisotropy data from ChREBP titration to a preformed complex of 14-3-3γ and FITC-labelled 2d and 2c. (e) Fluorescence anisotropy data from ChREBP titration to FITC-labelled 2d and 2c (f) 2D fluorescence anisotropy data of 14-3-3γ titration to FITC-labelled 2d (20 nM) with varying ChREBP peptide concentrations (*n* = 2). (g) Bar plot representation of obtained apparent *K*_D_ values in the 2D fluorescence anisotropy assay. The calculated *K*^II^_D_ and cooperativity factor α from a thermodynamic model are given. (h) Fluorescence anisotropy based selectivity study in which eight partner peptides of 14-3-3 are titrated to a preformed complex of 14-3-3γ (2 μM) and FITC-labelled 2d (20 nM) showing strong selectivity for 14-3-3/ChREBP stabilization (*n* = 2).

Interestingly, where most 14-3-3 binders are phosphorylated, ChREBP binds to 14-3-3 in a phosphorylation-independent manner and in a unique alpha-helical conformation (Fig. 5a).^20^ Since ChREBP does not engage with the phospho-binding pocket, this leaves a critical hotspot region within the 14-3-3 groove unoccupied. In contrast, N-terminally truncated peptide 2d does engage this pocket with a phosphoserine and binds uniquely *via* an N-terminal motif in the 14-3-3 binding groove (Fig. 5b).

A structural overlay of 14-3-3 bound to ChREBP and 14-3-3 bound to 2d clearly shows the complementary binding of the two peptides within the 14-3-3 binding groove (Fig. 5c). The phosphoserine of 2d occupies a composite pocket formed by the phospho-binding pocket of 14-3-3 and the α-helix of ChREBP. Similar to AMP,^47^ potentially 2d forms hydrogen bonds and electrostatic interactions with both 14-3-3 and ChREBP, leading to cooperative ternary complex formation of 14-3-3/ChREBP/2d (ESI Fig. S13†).

The cooperative protein complex formation of 14-3-3/ChREBP/2d was studied by titrating ChREBP to a complex of 14-3-3 and FITC-labelled 2d in a FA assay (Fig. 5d). Analysis of the assay showed a dose-dependent increase in 14-3-3/ChREBP/2d complex formation with increasing concentrations of ChREBP, with a half maximal ternary complex concentration (CC_50_) of 1.4 ± 0.5 μM. This increase in anisotropy was not observed when titrating ChREBP to FITC-labelled 2d alone (Fig. 5e). Furthermore, elongation of 2d with the −1 amino acid thiazolylalanine (peptide 2c), which was expected to clash with the ChREBP (ESI Fig. S14†), prevents the ternary complex formation as observed by the lack of increase in anisotropy values (Fig. 5d).

To quantify the cooperativity behaviour of ternary protein complex formation, we conducted a 2D titration of 14-3-3 to FITC-labelled 2d in the presence of a varied but constant concentration of ChREBP in a dose-dependent manner (Fig. 5f). From the 2D titrations, we determined the binding affinity (*K*_D_) for each concentration of ChREBP (Fig. 5g). Analysis showed a strong increase in binding affinity between 14-3-3 and 2d upon increasing concentrations of ChREBP. For the binding curves at the three highest concentrations of ChREBP peptide (Fig. 5f), we observe an increase in anisotropy levels for the upper plateau, which we hypothesized might be caused by the increase in the molecular volume of the complex upon ChREBP binding. Furthermore, these curves show a decrease again at higher 14-3-3 concentrations, indicating a decrease in molecular volume. For these data points 14-3-3 exceeds the peptide concentrations (20 nM FITC-2d; 0.01–10 μM ChREBP) which encourages more binary complex formation between 14-3-3 and one of the two peptides rather than ternary complex formation.^48,49^ Determination of the *K*_D_ for these concentrations is therefore done using only data points up to the highest anisotropy levels.

To accurately determine the cooperativity factor (*α*) of ternary 14-3-3/ChREBP/2d complex formation we fitted 2D titration data to a thermodynamic equilibrium model (ESI Fig. S15†).^50^ This model describes thermodynamic equilibrium constants of multicomponent systems. Cooperativity analysis of the complex elicited an α-factor of 36, indicating strong cooperative ternary complex formation. The model furthermore independently determined the apparent binding affinity of the ChREBP protein (*K*^II^_D_) based on the 2D titrations to be 1.6 μM which corresponds with the affinity of ChREBP for 14-3-3 as found in earlier studies.^20^

We hypothesize that the enhanced cooperative of the ternary complex formation is a function of electrostatic and hydrogen bonding interactions at the interface of the ternary 14-3-3/ChREPB/2d complex. Whilst, significant efforts we are made to crystalize the ternary complex, it was unsuccessful. Nonetheless, the analogs binding mode of 2d compared with AMP strongly supports this conclusion. Notably, the mechanism of cooperative ternary 14-3-3/ChREBP/2d complex formation is dependent on the presence of 14-3-3. The ternary complex formation proceeds *via* two-step binding mechanism where either 2d or ChREBP binds first 14-3-3, and the addition of the third interaction partner enhances the apparent avidity of the protein complex (ESI Fig. S15†).

The development of a molecular glue for the hub protein 14-3-3 is accompanied by the challenge of selectivity. To investigate the selective stabilization of the 14-3-3/ChREBP interaction, peptide 2d was screened against a panel of known 14-3-3 partner proteins with differing binding modes and affinities (Fig. 5h and ESI Table S7†). The panel consisted of seven peptides representing the F-domain of Estrogen Receptor α (ERα), Son Of Sevenless homolog 1 (SOS1), serine/threonine-protein kinase B-Raf, estrogen-related receptor gamma (ERRγ), Ubiquitin carboxyl-terminal hydrolase 8 (USP8), Peptidyl-prolyl *cis*–*trans* isomerase NIMA-interacting 1 (PIN1) and the p65 subdomain of NF-κB. Analysis of the peptide screen showed highly selective ternary complex formation of 14-3-3/peptide 2d/ChREBP. All other partner proteins showed a negative cooperative complex formation, inhibiting the formation of the binnary 14-3-3/2d protein complex formation, at significantly higher client peptide concentrations as compared to the cooperative stabilizing 14-3-3/ChREBP/2d system (Fig. 5h).

## Conclusions

To date there are limited reports of *de novo* development of molecular glues that target native PPIs, largely due to a lack of information on how to navigate the molecular glue drug development landscape for protein complexes. Functional mapping of a native protein complex provides valuable insight into protein complex formation and, similar to PPI inhibition,^25,31^ can be exploited to effectively triage molecular glue drug development.

Here we applied functional mapping to the 14-3-3/peptide 1 complex providing mechanistic insight into 14-3-3 client protein binding. We identified three critical hotspots in the 14-3-3 binding groove, additional to the phosphate binding pocket (Fig. 4d(i–iii)). First, Asn226 engages in hydrogen bond interactions with the backbone heavy atoms of its binding partner. Additionally, the hydrophobic patch formed by residues Leu218, Ile219 and Leu222 of 14-3-3 showed to contribute significantly to binding. Finally, electrostatic targeting of Lys122 showed to dominate the binding energy between 14-3-3 and peptide 1, making Lys122 a clear hotspot within the 14-3-3 binding groove.

Utilizing the functional mapping results, we developed a minimum peptide sequence (2d) that was able to selectively stabilize the 14-3-3/ChREBP PPI with an attractive cooperativity factor (*α*) of 36. Tool peptide 2d stands as a valuable proof of concept for 14-3-3 molecular glue design based on functional mapping of 14-3-3 hotspots.

Hotspot identification is not only valuable for the *de novo* design of molecular glues but can also be key in the improvement of already identified molecular glues. Structural analysis of known molecular glues for various 14-3-3 PPIs, among which pyrrolidone ligands, imine- and disulfide-tethered fragments, shows the proximity of these ligands to hotspots Lys122 and the hydrophobic patch formed by Leu218, Ile219 and Leu222 (ESI Fig. S7†).^18,35–37,46^ Therefore, enhancing cooperative binding of these molecular glues, by extrapolating the hotspot information into hit-to-lead optimization, could easily be envisioned. This hypothesis is confirmed upon structural analysis of the pyrrolidone compounds which contain a nitro-group which engages with Lys122 in a similar manner to peptide 1, allowing PPI stabilization of both 14-3-3/ERα and 14-3-3/CaMKK2 complexes.^46^ This example shows that specific targeting of hotspot residues in 14-3-3 is important in the design of PPI stabilizers. Introduction of such specific moieties to target 14-3-3 hotspots into early stage hit fragments, such as the disulfide fragments identified as 14-3-3/ERα and 14-3-3/ERRγ PPI stabilizers.^35,36^ could strongly increase their stabilization effects.

Functional mapping is also particularly interesting for more therapeutically relevant glues and cooperative PROTACs such as IMiDs^51^ and AT1,^52^ respectively. This approach would enable the triage of specific amino acid residues for small molecule optimization, particularly towards selectivity. For instance, functional mapping of the ternary complex between CRBN/Thalidomide/SALL4 ^51^ would provide valuable information regarding critical amino acids to target within the loop regions of the SALL4 zinc finger. Notably, given CRBN does not natively interact with SALL4, these experiments would need to be performed in the presence of thalidomide. This presents an opportunity to also observe critical interactions with thalidomide, which in turn leads to cooperative complex formation.

To conclude, this work shows that functional mapping of hotspots provides a triage map to orthosteric molecular glue drug design and optimization. Specifically, this study shows how hotspot information can be exploited to identify and optimize weak affinity and attractive cooperative chemical matter.

## Data availability

Crystal structures described in this manuscript have been deposited to the PDB (7ZMU & 7ZMW).

## Author contributions

B. A. S, L. B., C. O. and P. J. C. conceptualized and initiated the project. C. O., P. J. C., and L. B. supervised the project and provided scientific guidance. F. W. B. C., and W.-H. L, performed peptide synthesis and purification. B. A. S., F. W. B. C., and W.-H. L performed FA-based studies and energy contribution analysis. W.-H. L., and A. A. K. performed ITC experiments and data analysis. B. A. S., F. W. B. C., M. A. M. P. and E. J. V performed and analysed PPI stabilization assays. B. A. S., and P. J. C. wrote the manuscript with essential input and feedback from all other authors.

## Conflicts of interest

The authors declare the following competing financial interest(s): L. B. and C. O. are scientific co-founders of Ambagon Therapeutics.

## Supplementary Material

SC-013-D2SC04662H-s001
