# Supplementary material for: Functional mapping of the 14-3-3 hub protein as a guide to design 14-3-3 molecular glues
Source: Chem Sci. 2022 Oct 25;13(44):13122–31. doi: 10.1039/d2sc04662h (PMC9667936; doi:10.1039/d2sc04662h)
Supplement: SC-013-D2SC04662H-s001 [file SC-013-D2SC04662H-s001.pdf]

Electronic Supplementary Material (ESI) for Chemical Science.
This journal is © The Royal Society of Chemistry 2022

Supplementary Information for

Functional mapping of the 14-3-3 hub protein as a guide to design 14-3-3 molecular glues

Bente A. Somsen, Fenna W. B. Craenmehr, Wei-Hong W. Liu, Auke A. Koops, Marloes A.M. Pennings, Emira J. Visser, Christian Ottmann, Peter J. Cossar[1] and Luc Brunsveld[1]

[1] Laboratory of Chemical Biology, Department of Biomedical Engineering and Institute for Complex Molecular Systems, Eindhoven University of Technology, P.O. Box 513, 5600 MB Eindhoven, The Netherlands

*Peter J. Cossar and Luc Brunsveld

Email: p.cossar@tue.nl and l.brunsveld@tue.nl

This PDF file includes:

Figures S1 to S15
Tables S1 to S8
Experimental procedures

a.

Construct	Sequence										ALC (%)
**14-3-3.1**	D-Leu	Nph	β-Ser	Nph	pSer	Nph	β-Ser	β-Ala	Nph	Lys	99
**14-3-3.2**	Fph	D-Val	Nph	Fph	pSer	Cpa	D-Gln	Fph	Fph	Lys	99
**14-3-3.3**	Fph	Fph	Hyp	Fph	pSer	Thz	β-Ser	β-Ala	Nph	Lys	97
**14-3-3.4**	Cha	Cpa	β-Ser	Thz	pSer	Cha	β-Ser	Nph	β-Ala	Lys	91
**14-3-3.5**	Cha	Cha	β-Ser	Fph	pSer	Thz	β-Ser	β-Ala	Hyp	Lys	91
**14-3-3.6**	Cha	Cha	β-Ser	Orn	pSer	Nph	β-Ser	β-Ser	Nph	Lys	90
**14-3-3.7**	Cha	Nph	Hyp	Fph	pSer	Thz	β-Thr	β-Ala	Nph	Lys	85
**14-3-3.8**	Cha	Fph	β-Ser	Cpa	pSer	β-Thr	Nph	β-Ala	Nph	Lys	85
**14-3-3.9**	Thz	Thz	Hyp	Thz	pSer	Nph	β-Ser	β-Ser	Nph	Lys	85
**14-3-3.10**	Cha	β-Ala	Nph	Aph	pSer	D-Val	Aad	D-Gln	Nph	Lys	85
**14-3-3.11**	D-Gln	Cpa	Nph	Cpa	pSer	Fph	β-Ser	β-Ala	Nph	Lys	84
**14-3-3.12**	D-Lys	D-Val	Nph	Thz	pSer	Nph	β-Ser	β-Ala	Nph	Lys	84
**14-3-3.13**	Fph	β-Ser	Hyp	Thz	pSer	Nph	β-Ser	β-Ser	Nph	Lys	83
**14-3-3.14**	Cha	Cpa	β-Ser	Orn	pSer	Fph	Cpa	β-Ser	Nph	Lys	82
**14-3-3.15**	Cha	β-Ala	β-Ser	Cpa	pSer	Fph	β-Ala	β-Ala	Nph	Lys	81
**14-3-3.16**	Cpa	Fph	Cha	Thz	pSer	Thz	Hyp	Aph	Hyp	Lys	81
**14-3-3.17**	Cha	Fph	β-Ser	Cpa	pSer	Aph	β-Ser	Aad	Fph	Lys	81

b.

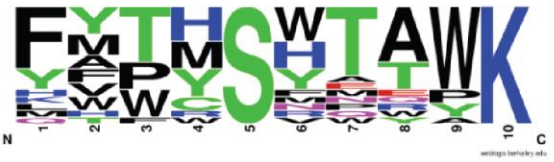

A = β-alanine; C = ornithine; D = D-aspartate; E-aminoadipic acid; F = cyclohexyl alanine; G = diaminobutyric acid; H= thiazolyl alanine; K = D-lysine (pos 1-9) or L-lysine (pos. 10). L = D-leucine; M = cyclo-propylalanine; N = β-homothreonine; P = hydroxyproline; Q = D-glutamine; R = 4-aminophenylalanine; S = phosphoserine; T = β-homoserine; V = norvaline; W = 4-nitrophenylalalnine; Y = 4-fluorophenylalanine

Figure S1. Non-natural peptide screening results. (a) Table of seventeen obtained hit sequences from the non-natural peptide library screen. (b) Positional frequency analysis of identified 14-3-3γ binding hits. Each letter represents a non-natural amino acid and the size of the letter determines its abundance in the hit sequences. Figures taken from Quartararo (2020).

Table S1. Binding characteristics peptide **1**. K_D and ΔG values obtained from fluorescence anisotropy (FA) assays and K_D, ΔG, ΔH and -TΔS data obtained from isothermal titration calorimetry (ITC) experiments. Values are given for all measured replicates (3x for FA; 2x for ITC). N.A. = not available.

**Technique**	**FA**	**FA**	**ITC**	**ITC**	**ITC**	**ITC**
	**K_D (M)**	**ΔG**	**K_D (M)**	**ΔG**	**ΔH**	**−TΔS**
**Replicate 1**	1.64E-08	-10.6	4.47E-08	-10.0	-11.0	0.9
**Replicate 2**	1.81E-08	-10.6	4.53E-08	-10.0	-10.5	0.4
**Replicate 3**	2.33E-08	-10.4	N.A.	N.A.	N.A.	N.A.
**Mean**	1.92E-08	-10.5	4.50E-08	-10.0	-10.7	0.7
**Std. deviation**	3.56E-09	0.1	4.17E-10	0.0	0.4	0.4

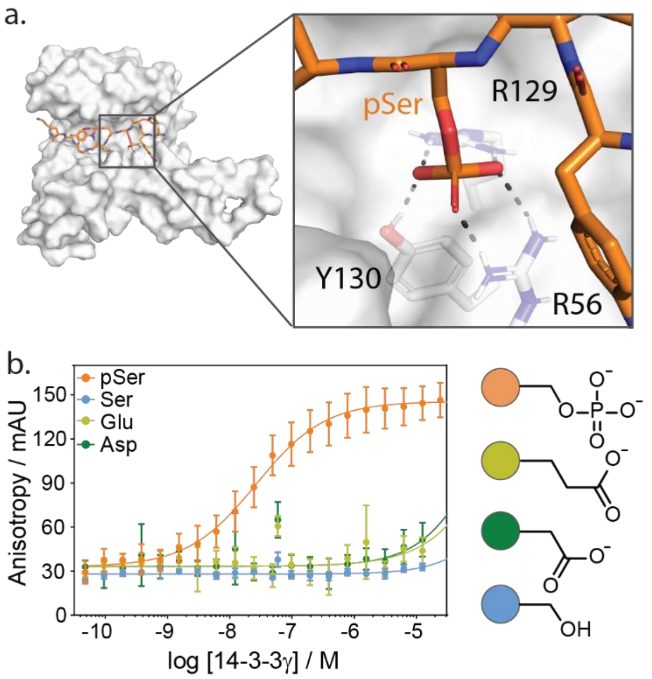

Figure S2. Phosphoserine replacement study. (a) Crystal structure of 14-3-3σ (white surface) bound to the peptide **1** (orange sticks). Enlarged view of the electrostatic interaction between the phosphoserine residue of the **1** and the 14-3-3 phospho-accepting pocket (R129, R56, and Y130). (b) Fluorescence anisotropy assay of 14-3-3σ titration to fixed concentrations fluorescein-labelled peptide (10 nM) and schematic representation of phosphoserine containing peptide (orange) and the tested phosphoserine replacements being serine (blue), glutamic acid (yellow) and aspartic acid (green).

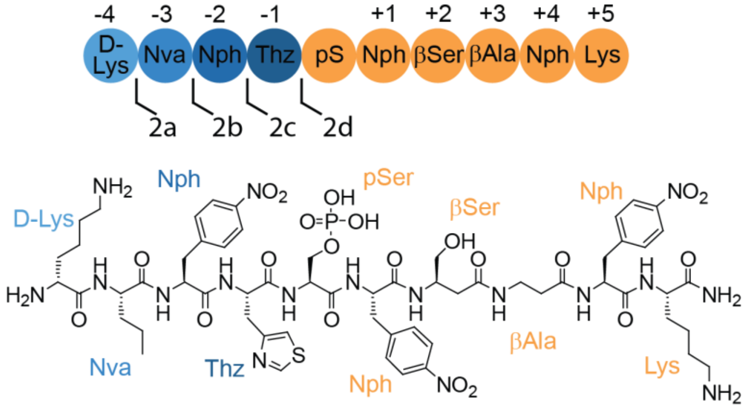

	-4	-3	-2	-1		+1	+2	+3	+4	+5
1	D-Lys	Nva	Nph	Thz	pSer	Nph	βSer	βAla	Nph	Lys
2a		Nva	Nph	Thz	pSer	Nph	βSer	βAla	Nph	Lys
2b			Nph	Thz	pSer	Nph	βSer	βAla	Nph	Lys
2c				Thz	pSer	Nph	βSer	βAla	Nph	Lys
2d					pSer	Nph	βSer	βAla	Nph	Lys

Figure S3. Sequences N-terminal truncated peptides 2a-d. Schematic representation and chemical structure of peptide **1** and the N-terminal truncations. Within these peptides we stepwise remove the N-terminal amino acids leading to peptides **2a-d**.

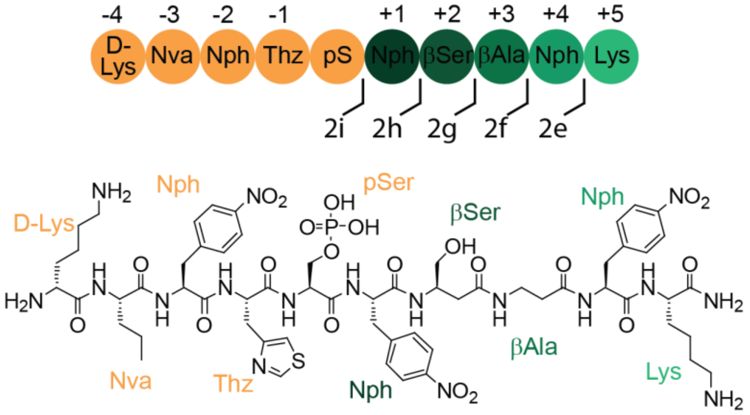

	-4	-3	-2	-1		+1	+2	+3	+4	+5
1	D-Lys	Nva	Nph	Thz	pSer	Nph	βSer	βAla	Nph	Lys
2e	D-Lys	Nva	Nph	Thz	pSer	Nph	βSer	βAla	Nph	
2f	D-Lys	Nva	Nph	Thz	pSer	Nph	βSer	βAla		
2g	D-Lys	Nva	Nph	Thz	pSer	Nph	βSer			
2h	D-Lys	Nva	Nph	Thz	pSer	Nph				
2i	D-Lys	Nva	Nph	Thz	pSer					

Figure S4. Sequences C-terminal truncated peptides 2e-i. Schematic representation and chemical structure of peptide **1** and the C-terminal truncations. Within these peptides we stepwise remove the C-terminal amino acids leading to peptides **2e-i**.

Table S2. Overview K_D and ΔΔG analysis N-terminal truncated peptides 2a-2d. K_d values are determined from the FA-based binding studies of 14-3-3 to each of the peptides. The fold change in binding affinity is determined by dividing the K_D from a peptide with the K_D of the previous (one amino acid longer) peptide. Furthermore the K_D is transformed into ΔG values which in the end is transformed into a ΔΔG analysis by subtracting the ΔG from each peptide with the ΔG from the previous (one amino acid longer) peptide.

**Affinity K_D (M)**	**1**	**2a**	**2b**	**2c**	**2d**	
**Removal**	**-**	**-4 D-Lys**	**-3 Nva**	**-2 NPh**	**-1 Thz**	
**Replicate 1**	1,64E-08	2,83E-08	1,03E-07	5,21E-08	2,12E-06	
**Replicate 2**	1,81E-08	4,63E-08	1,08E-07	5,68E-08	2,60E-06	
**Replicate 3**	2,33E-08	3,86E-08	1,01E-07	5,63E-08	2,43E-06	
**Mean**	1,92E-08	3,77E-08	1,04E-07	5,51E-08	2,39E-06	
**Std. deviation**	3,56E-09	9,07E-09	3,44E-09	2,59E-09	2,45E-07	
**Fold change to previous**	**1**	**2a**	**2b**	**2c**	**2d**	
**Removal**	**-**	**-4 D-Lys**	**-3 Nva**	**-2 NPh**	**-1 Thz**	
**Replicate 1**		1,72	3,64	0,51	40,69	
**Replicate 2**		2,56	2,32	0,53	45,81	
**Replicate 3**		1,66	2,61	0,56	43,18	
**Mean**		1,98	2,86	0,53	43,23	
**Std. deviation**		0,50	0,69	0,03	2,56	
**ΔG (kcal/mol)**	**1**	**2a**	**2b**	**2c**	**2d**	
**Removal**	**-**	**-4 D-Lys**	**-3 Nva**	**-2 NPh**	**-1 Thz**	
**Replicate 1**	-10,61	-10,29	-9,53	-9,93	-7,74	
**Replicate 2**	-10,56	-10,00	-9,50	-9,88	-7,61	
**Replicate 3**	-10,41	-10,11	-9,54	-9,88	-7,65	
**Mean**	-10,53	-10,13	-9,52	-9,90	-7,67	
**Std. deviation**	0,11	0,15	0,02	0,03	0,06	
**ΔΔG (kcal/mol) to previous**	**1**	**2a**	**2b**	**2c**	**2d**	**N-term**
**Removal**	**-**	**-4 D-Lys**	**-3 Nva**	**-2 NPh**	**-1 Thz**	
**Replicate 1**		0,32	0,76	-0,40	2,19	2,88
**Replicate 2**		0,56	0,50	-0,38	2,26	2,94
**Replicate 3**		0,30	0,57	-0,34	2,23	2,75
**Mean**		0,39	0,61	-0,37	2,23	2,86
**Std. deviation**		0,14	0,14	0,03	0,04	0,10

Table S3. Overview K_D and ΔΔG analysis C-terminal truncated peptides 2e-2i. K_D values are determined from the FA-based binding studies of 14-3-3 to each of the peptides. The fold change in binding affinity is determined by dividing the K_D from a peptide with the K_D of the previous (one amino acid longer) peptide. Furthermore the K_D is transformed into ΔG values which in the end is transformed into a ΔΔG analysis by subtracting the ΔG from each peptide with the ΔG from the previous (one amino acid longer) peptide.

**Affinity Kd (M)**	**1**	**2e**	**2f**	**2g**	**2h**	**2i**	
**Removal**	**-**	**+5 Lys**	**+4 Nph**	**+3 bAla**	**+2 bSer**	**+1 Nph**	
**Replicate 1**	1,64E-08	9,09E-08	1,15E-06	2,22E-06	6,46E-06	2,73E-05	
**Replicate 2**	1,81E-08	1,29E-07	1,49E-06	2,73E-06	7,29E-06	2,59E-05	
**Replicate 3**	2,33E-08	9,25E-08	1,20E-06	2,14E-06	6,83E-06	2,96E-05	
**Mean**	1,92E-08	1,04E-07	1,28E-06	2,37E-06	6,86E-06	2,76E-05	
**Std. deviation**	2,91E-09	1,75E-08	1,47E-07	2,63E-07	3,41E-07	1,51E-06	
**Fold change to previous**	**1**	**2e**	**2f**	**2g**	**2h**	**2i**	
**Removal**	**-**	**+5 Lys**	**+4 Nph**	**+3 bAla**	**+2 bSer**	**+1 Nph**	
**Replicate 1**		5,54	12,69	1,92	2,91	4,23	
**Replicate 2**		7,13	11,53	1,84	2,67	3,56	
**Replicate 3**		3,98	12,93	1,79	3,19	4,33	
**Mean**		5,55	12,39	1,85	2,92	4,04	
**Std. deviation**		1,57	0,75	0,07	0,26	0,42	
**ΔG (kcal/mol)**	**1**	**2e**	**2f**	**2g**	**2h**	**2i**	
**Removal**	**-**	**+5 Lys**	**+4 Nph**	**+3 bAla**	**+2 bSer**	**+1 Nph**	
**Replicate 1**	-10,61	-9,60	-8,10	-7,71	-7,08	-6,22	
**Replicate 2**	-10,56	-9,39	-7,95	-7,59	-7,00	-6,25	
**Replicate 3**	-10,41	-9,59	-8,07	-7,73	-7,04	-6,18	
**Mean**	-10,53	-9,53	-8,04	-7,67	-7,04	-6,22	
**Std. deviation**	0,11	0,12	0,08	0,08	0,04	0,04	
**ΔΔG (kcal/mol)**	**1**	**2e**	**2f**	**2g**	**2h**	**2i**	**C-term**
**Removal**	**-**	**+5 Lys**	**+4 Nph**	**+3 bAla**	**+2 bSer**	**+1 Nph**	
**Replicate 1**		1,01	1,50	0,39	0,63	0,85	4,39
**Replicate 2**		1,16	1,45	0,36	0,58	0,75	4,30
**Replicate 3**		0,82	1,52	0,34	0,69	0,87	4,23
**Mean**		1,00	1,49	0,36	0,63	0,82	4,31
**Std. deviation**		0,17	0,04	0,02	0,05	0,06	0,08

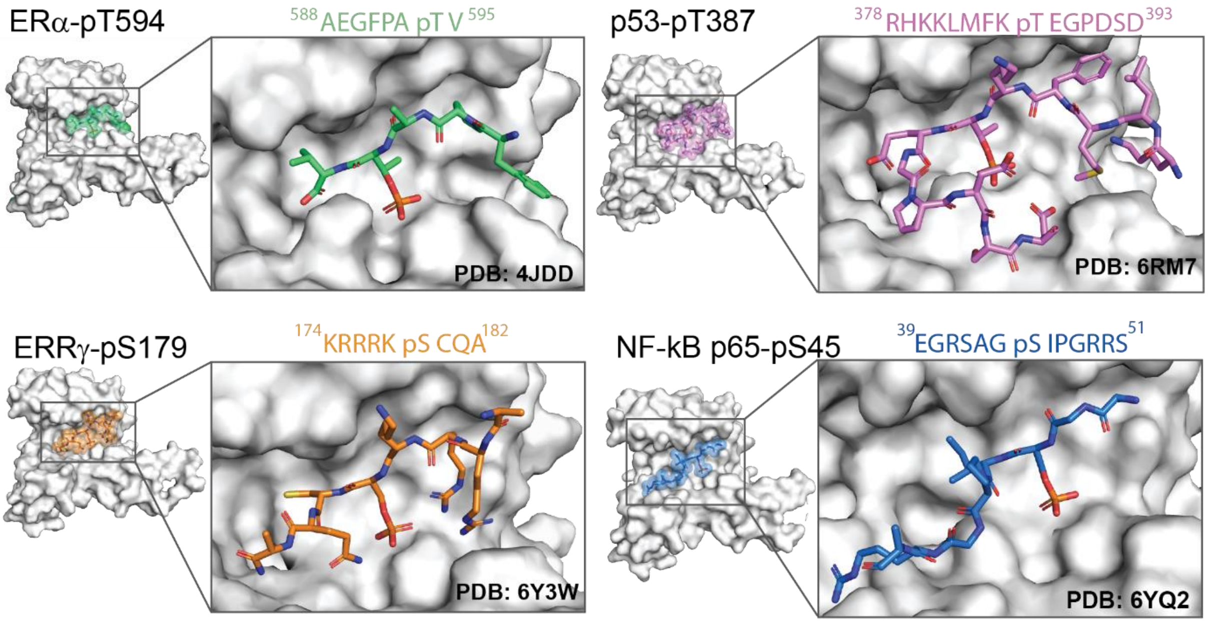

Figure S5. Examples of 14-3-3 binders. Crystal structures of 14-3-3σ (white surface) bound to four different phosphorylated peptides (stick representations); Estrogen Receptor alpha (ERα), p53, Estrogen Related Receptor gamma (ERRγ) and NF-kB p65. Crystal structures represent that most peptides bind within their N-terminal side (right from the phosphoserine) into the binding groove, whereas the C-terminal is often lacking or binding outside the binding groove.

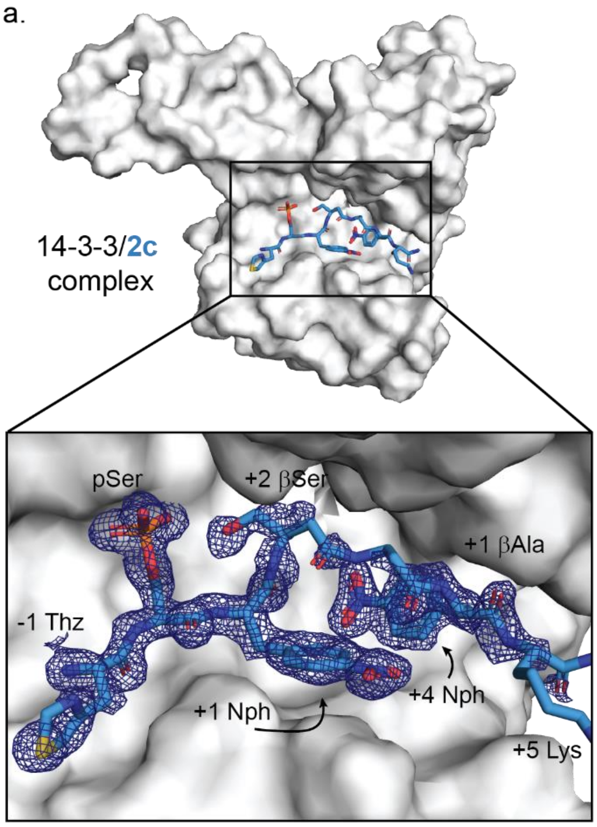

Figure S6. Crystal structures of N-terminal truncated peptides 2c-d. (a/b) Structure of 14-3-3 (white surface) bound to either of the N-terminally truncated peptides (blue sticks). A total view and an enlarged view of the peptide binding. The final 2Fo-Fc electron density map is represented as blue mesh contoured at 1σ. PDB: 7ZMU & 7ZMW

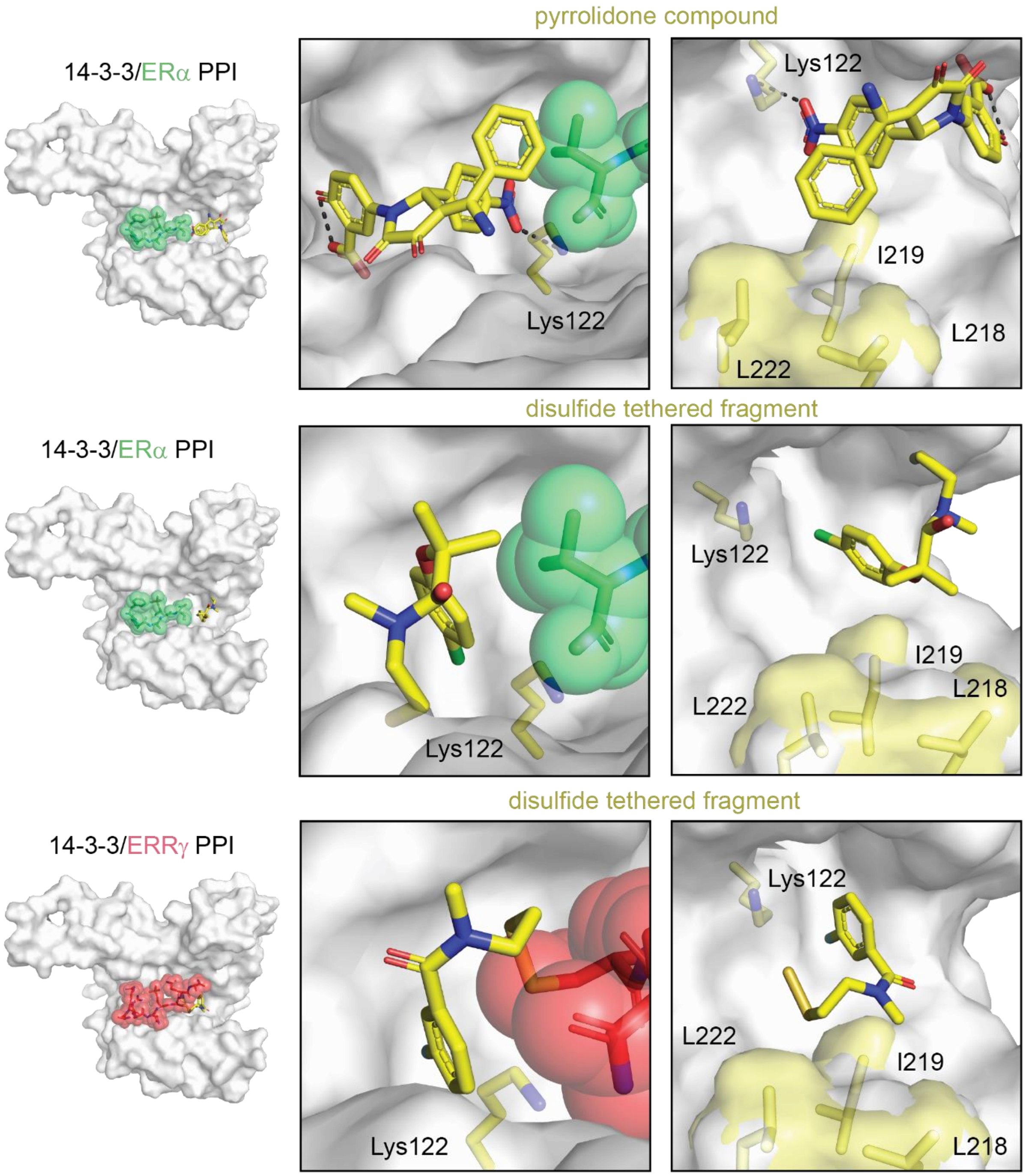

Figure S7a. Crystal structures of known PPI stabilizing compounds. Two different 14-3-3 PPIs, namely 14-3-3/ERα (PDB: 6TL3 & 6HHP), 14-3-3/ERRγ (PDB: 6Y3W), in complex with a pyrrolidone compounds or disulfide tethered fragments (yellow sticks) that stabilizes the PPI complex. A total overview of 14-3-3 (white surface) bound to peptide (reg/green spheres) and the stabilizer (yellow sticks) is shown at the left. A enlarged view is given in the middle panel of compound binding at the PPI interface. The right panel envisions the proximity of these compounds to hotspot residues K122 (yellow sticks) and the hydrophobic patch formed by L218, I219 and L222 (yellow sticks and surface)

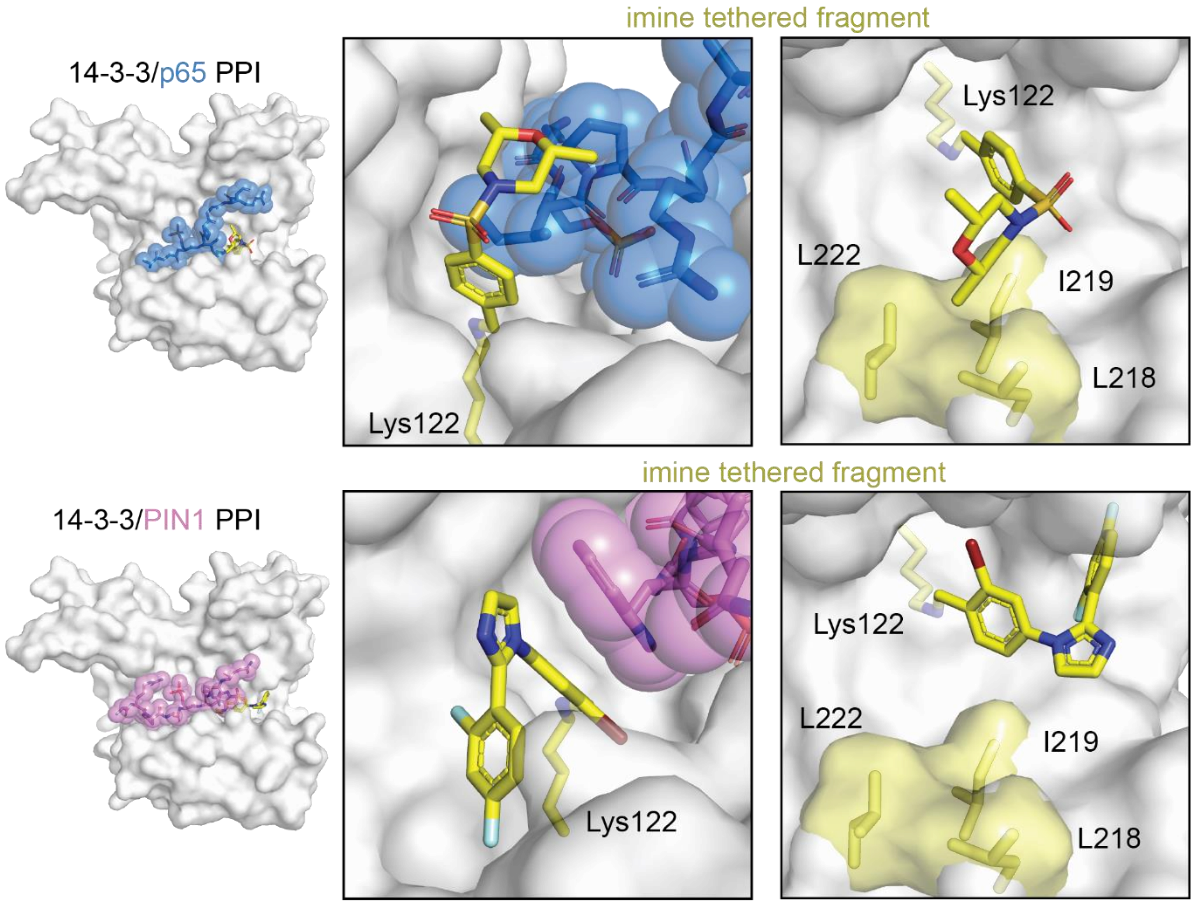

Figure S7b. Crystal structures of known PPI stabilizing compounds. Two different 14-3-3 PPIs, namely 14-3-3/p65 (PDB: 6YQ2), 14-3-3/PIN1 (PDB: 7BFW), in complex with a pyrrolidone compounds or disulfide tethered fragments (yellow sticks) that stabilizes the PPI complex. A total overview of 14-3-3 (white surface) bound to peptide (blue/pink spheres) and the stabilizer (yellow sticks) is shown at the left. A enlarged view is given in the middle panel of compound binding at the PPI interface. The right panel envisions the proximity of these compounds to hotspot residues K122 (yellow sticks) and the hydrophobic patch formed by L218, I219 and L222 (yellow sticks and surface).

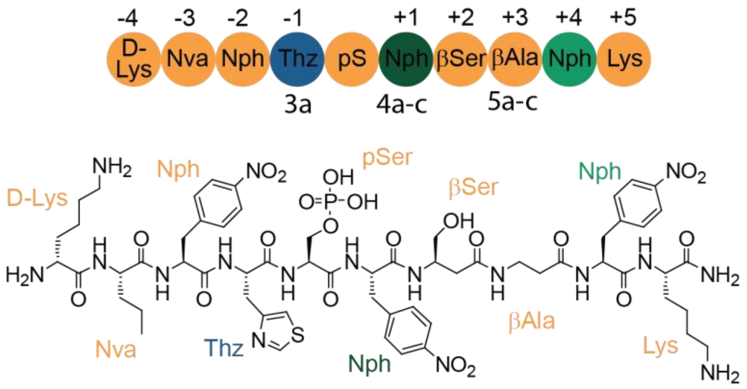

	-4	-3	-2	-1		+1	+2	+3	+4	+5
1	D-Lys	Nva	Nph	Thz	pSer	Nph	βSer	βAla	Nph	Lys
3a	D-Lys	Nva	Nph	Ala	pSer	Nph	βSer	βAla	Nph	Lys
4a	D-Lys	Nva	Nph	Thz	pSer	Tyr	βSer	βAla	Nph	Lys
4b	D-Lys	Nva	Nph	Thz	pSer	Phe	βSer	βAla	Nph	Lys
4c	D-Lys	Nva	Nph	Thz	pSer	Ala	βSer	βAla	Nph	Lys
5a	D-Lys	Nva	Nph	Thz	pSer	Nph	βSer	βAla	Tyr	Lys
5b	D-Lys	Nva	Nph	Thz	pSer	Nph	βSer	βAla	Phe	Lys
5c	D-Lys	Nva	Nph	Thz	pSer	Nph	βSer	βAla	Ala	Lys

Figure S8. Sequences point mutated peptides 3a, 4a-c, 5a-c. Schematic representation and chemical structure of peptide **1** and the point mutations. Within these peptides point mutations are made at position -1 (Thz) leading to peptide **3a**, position +1 (Nph) leading to peptides **4a-c**, and position +4 (Nph) leading to peptides **5a-c**. Mutations were made to alanine, tyrosine and phenylalanine residues.

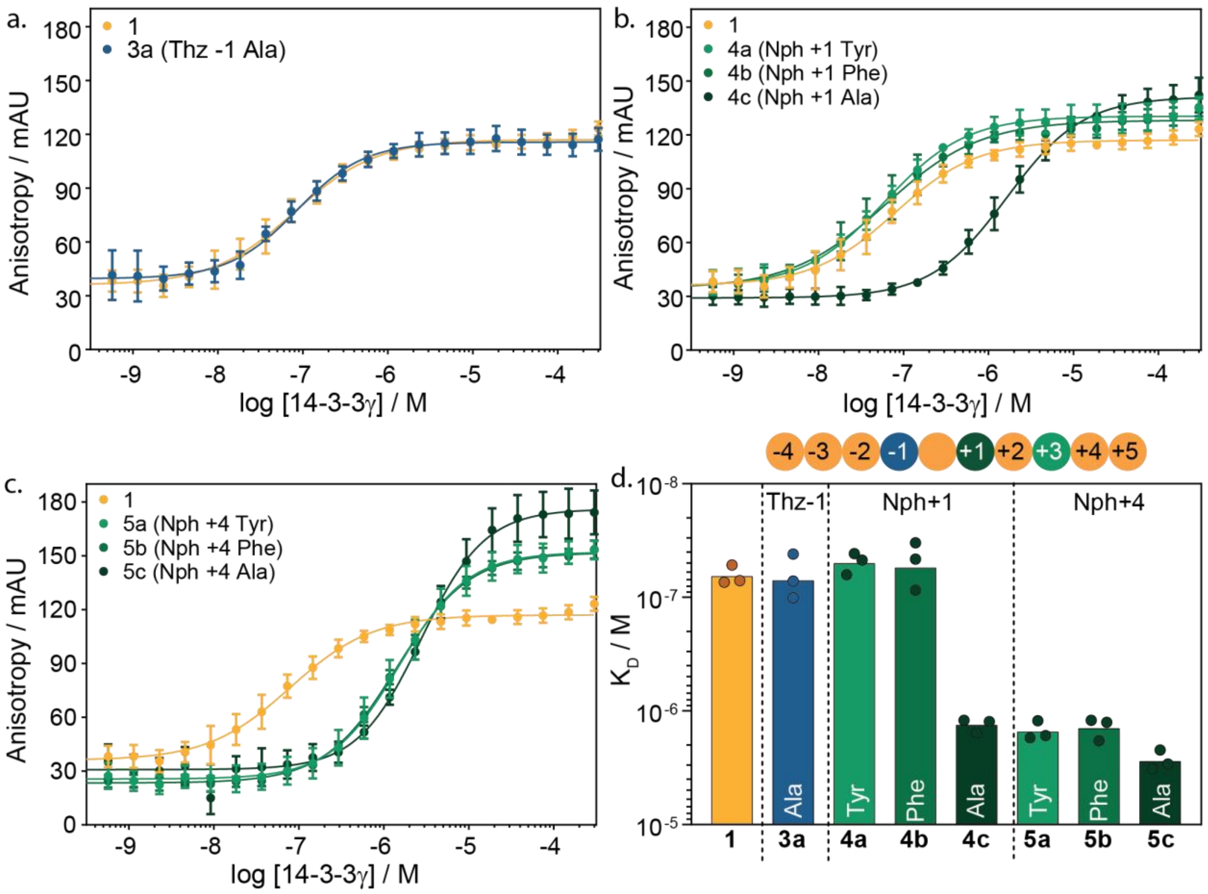

Figure S9. FA assay mutant peptides. (a) Fluorescence anisotropy assay of 14-3-3γ titration to a fixed concentration (10 nM) of FITC-labelled **1** (orange) and -1 mutated peptide (blue). (b) Fluorescence anisotropy assay of 14-3-3γ titration to a fixed concentration (10 nM) of FITC-labelled **1** (orange) and +1 mutated peptides (green). (c) Fluorescence anisotropy assay of 14-3-3γ titration to a fixed concentration (10 nM) of FITC-labelled **1** (orange) and +4 mutated peptides (green). (d) Bar plot representation from obtained binding affinities (K_D) of mutated peptides and the fold change in affinity between different constructs. All data is recorded in triplicate from three independent experiments. The K_D obtained from each of the independent experiments is shows as single point and the bar represents the mean K_D of these three datapoints.

Table S4. Overview K_d and ΔΔG analysis mutated peptides (FA-based assays). K_d values are determined from the FA-based binding studies of 14-3-3 to each of the peptides. The fold change in binding affinity is determined by dividing the K_d from a peptide with the K_d of **1**. Furthermore the K_d is transformed into ΔG values which in the end is transformed into a ΔΔG analysis by subtracting the ΔG from each peptide with the ΔG from **1**.

**Affinity Kd (M)**	**1**	**3a**	**4a**	**4b**	**4c**	**5a**	**5b**	**5c**
**Mutant**	**-**	**Nph+4Ala**	**Nph+1Tyr**	**Nph+1Phe**	**Nph+1Ala**	**Nph +4Tyr**	**Nph +4Phe**	**Nph +4Ala**
**Replicate 1**	5.2E-08	1.0E-07	4.7E-08	4.6E-08	1.2E-06	1.7E-06	1.3E-06	3.2E-06
**Replicate 2**	7.1E-08	4.2E-08	4.1E-08	3.3E-08	1.2E-06	1.2E-06	1.2E-06	2.2E-06
**Replicate 3**	7.4E-08	7.2E-08	6.3E-08	8.7E-08	1.6E-06	1.7E-06	1.8E-06	2.9E-06
**Mean**	6.6E-08	7.1E-08	5.0E-08	5.5E-08	1.3E-06	1.5E-06	1.4E-06	2.8E-06
**Std. deviation**	1.2E-08	3.0E-08	1.1E-08	2.8E-08	2.0E-07	2.7E-07	3.5E-07	5.2E-07
**Fold change to 1**	**1**	**3a**	**4a**	**4b**	**4c**	**5a**	**5b**	**5c**
**Mutant**	**-**	**Nph+4Ala**	**Nph+1Tyr**	**Nph+1Phe**	**Nph+1Ala**	**Nph +4Tyr**	**Nph +4Phe**	**Nph +4Ala**
**Replicate 1**		1.9	0.9	0.9	24.0	33.2	24.3	62.1
**Replicate 2**		0.6	0.6	0.5	17.0	17.1	16.9	31.0
**Replicate 3**		1.0	0.9	1.2	21.3	22.5	24.9	39.8
**Mean**		1.2	0.8	0.8	20.8	24.2	22.0	44.3
**Std. deviation**		0.7	0.2	0.4	3.5	8.2	4.4	16.0
**ΔG (kcal/mol)**	**1**	**3a**	**4a**	**4b**	**4c**	**5a**	**5b**	**5c**
**Mutant**	**-**	**Nph+4Ala**	**Nph+1Tyr**	**Nph+1Phe**	**Nph+1Ala**	**Nph +4Tyr**	**Nph +4Phe**	**Nph +4Ala**
**Replicate 1**	-9.9	-9.5	-10.0	-10.0	-8.1	-7.9	-8.0	-7.5
**Replicate 2**	-9.7	-10.1	-10.1	-10.2	-8.1	-8.1	-8.1	-7.7
**Replicate 3**	-9.7	-9.7	-9.8	-9.6	-7.9	-7.9	-7.8	-7.5
**Mean**	-9.8	-9.8	-10.0	-9.9	-8.0	-7.9	-8.0	-7.6
**Std. deviation**	0.1	0.3	0.1	0.3	0.1	0.1	0.1	0.1
**ΔΔG (kcal/mol) to 1**	**1**	**3a**	**4a**	**4b**	**4c**	**5a**	**5b**	**5c**
**Mutant**	**-**	**Nph+4Ala**	**Nph+1Tyr**	**Nph+1Phe**	**Nph+1Ala**	**Nph +4Tyr**	**Nph +4Phe**	**Nph +4Ala**
**Replicate 1**		0.4	-0.1	-0.1	1.9	2.1	1.9	2.4
**Replicate 2**		-0.3	-0.3	-0.5	1.7	1.7	1.7	2.0
**Replicate 3**		0.0	-0.1	0.1	1.8	1.8	1.9	2.2
**Mean**		0.0	-0.2	-0.1	1.8	1.9	1.8	2.2
**Std. deviation**		0.4	0.1	0.3	0.1	0.2	0.1	0.2

a.

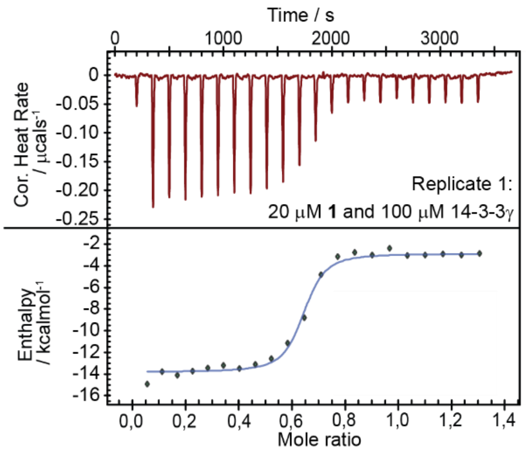

Model	Variable	Value	Confidence Interval	Confidence Level
Blank (constant)	blank (µcal)	-0,57367	±0,00000	95%
Independent	Kd (M)	4,469E-8	±2,435E-8	95%
	n	0,617	±0,011	95%
	ΔH (kcal/mol)	-10,96	±0,336	95%
	-TΔS (kcal/mol)	0,933		
	ΔG (kcal/mol)	-10,03		
	ΔS (cal/mol·K)	-3,128		

b.

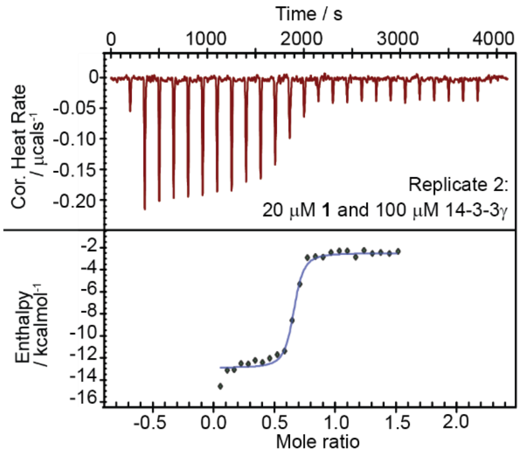

Model	Variable	Value	Confidence Interval	Confidence Level
Blank (constant)	blank (µcal)	-0,49702	±0,00000	95%
Independent	Kd (M)	4,529E-8	±2,740E-8	95%
	n	0,632	±0,013	95%
	ΔH (kcal/mol)	-10,45	±0,357	95%
	-TΔS (kcal/mol)	0,426		
	ΔG (kcal/mol)	-10,02		
	ΔS (cal/mol·K)	-1,430		

c.

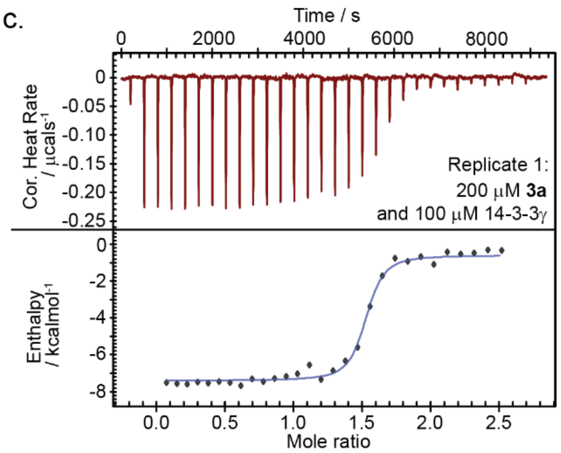

Model	Variable	Value	Confidence Interval	Confidence Level
Blank (constant)	blank (µcal)	-0,23888	±0,00000	95%
Independent	Kd (M)	6,965E-8	±3,035E-8	95%
	n	1,488	±0,016	95%
	ΔH (kcal/mol)	-6,809	±0,130	95%
	-TΔS (kcal/mol)	-2,955		
	ΔG (kcal/mol)	-9,764		
	ΔS (cal/mol·K)	9,912		

d.

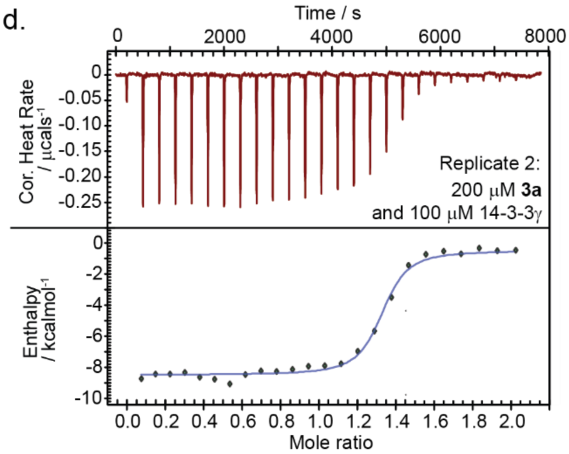

Model	Variable	Value	Confidence Interval	Confidence Level
Blank (constant)	blank (µcal)	-0,19675	±0,00000	95%
Independent	Kd (M)	8,857E-8	±3,220E-8	95%
	n	1,290	±0,013	95%
	ΔH (kcal/mol)	-7,998	±0,149	95%
	-TΔS (kcal/mol)	-1,624		
	ΔG (kcal/mol)	-9,622		
	ΔS (cal/mol·K)	5,447		

Figure S10A. Raw ITC data 1 and 3a. Measured raw thermograms, binding isotherms and derived thermodynamic values for 14-3-3γ binding to **1** and **3a** (Thz-1Ala). Used concentrations are state in the graph. A constant blank model is used to correct for heat of injection and independent model is used to model peptide binding (blue line). From this the thermodynamic parameters are determined as noted in each table associated with the thermograms. Each peptide is measured in two independent experiments (replicate 1 and 2) which are both shown here. **For thermodynamic parameters refer to Table S6.

a.

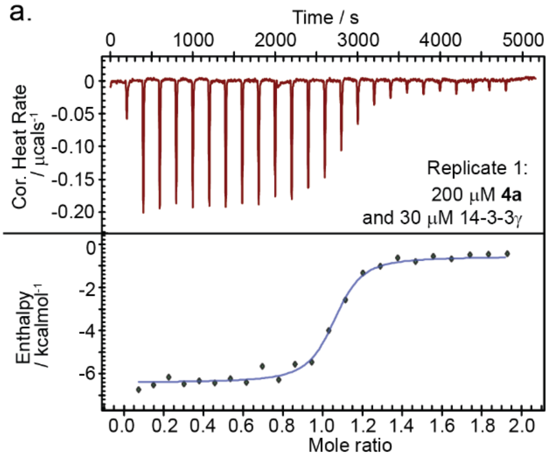

Model	Variable	Value	Confidence Interval	Confidence Level
Blank (constant)	blank (μcal)	-0,23025	±0,00000	95%
Independent	Kd (M)	1,263E-7	±5,216E-8	95%
	n	1,023	±0,016	95%
	ΔH (kcal/mol)	-5,830	±0,142	95%
	-TΔS (kcal/mol)	-3,582		
	ΔG (kcal/mol)	-9,411		
	ΔS (cal/mol·K)	12,01		

b.

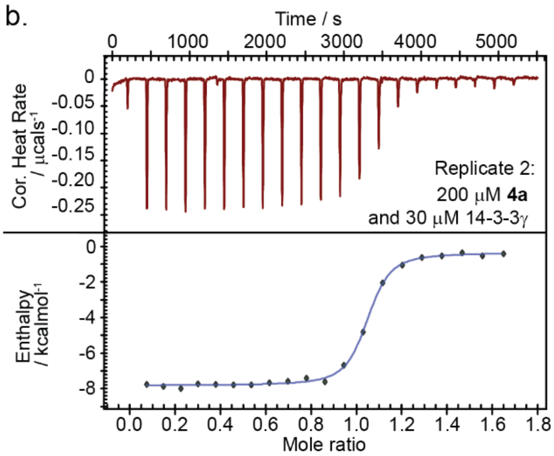

Model	Variable	Value	Confidence Interval	Confidence Level
Blank (constant)	blank (μcal)	-0,14833	±0,04404	95%
Independent	Kd (M)	6,090E-8	±1,408E-8	95%
	n	1,010	±0,006	95%
	ΔH (kcal/mol)	-7,450	±0,145	95%
	-TΔS (kcal/mol)	-2,393		
	ΔG (kcal/mol)	-9,844		
	ΔS (cal/mol·K)	8,027		

c.

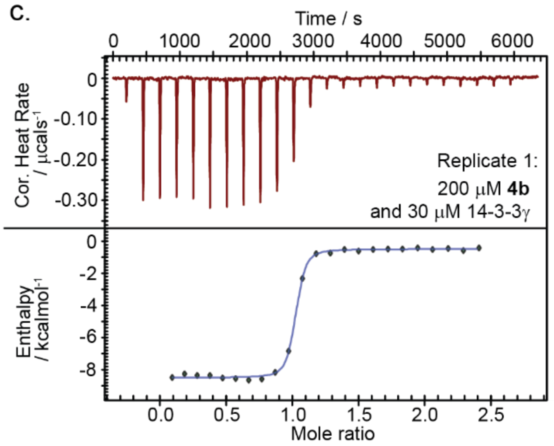

Model	Variable	Value	Confidence Interval	Confidence Level
Blank (constant)	blank (μcal)	-0,23907	±0,03212	95%
Independent	Kd (M)	2,622E-8	±6,824E-9	95%
	n	0,978	±0,005	95%
	ΔH (kcal/mol)	-8,024	±0,107	95%
	-TΔS (kcal/mol)	-2,319		
	ΔG (kcal/mol)	-10,34		
	ΔS (cal/mol·K)	7,777		

d.

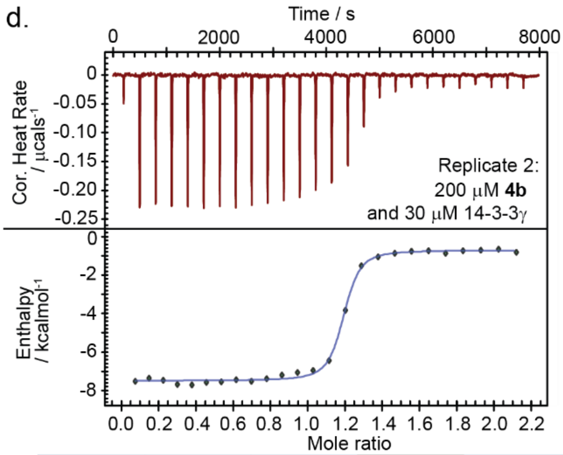

Model	Variable	Value	Confidence Interval	Confidence Level
Blank (constant)	blank (μcal)	-0,28345	±0,03429	95%
Independent	Kd (M)	3,764E-8	±1,115E-8	95%
	n	1,153	±0,006	95%
	ΔH (kcal/mol)	-6,783	±0,121	95%
	ΔS (cal/mol·K)	11,22		

Figure S10B. Raw ITC data 4a and 4b. Measured raw thermograms, binding isotherms and derived thermodynamic values for 14-3-3γ binding to **4a** (Nph+1Tyr) and **4b** (Nph+1Phe). Used concentrations are state in the graph. A constant blank model is used to correct for heat of injection and independent model is used to model peptide binding (blue line). From this the thermodynamic parameters are determined as noted in each table associated with the thermograms. Each peptide is measured in two independent experiments (replicate 1 and 2) which are both shown here. **For thermodynamic parameters refer to Table S6.

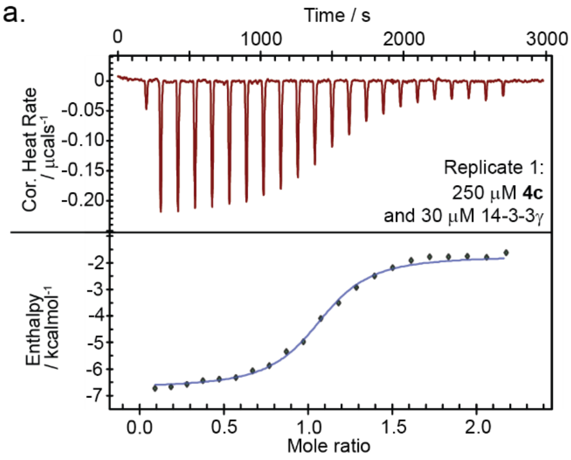

Model	Variable	Value	Confidence Interval	Confidence Level
Blank (constant)	blank (µcal)	-0,36754	±0,00000	95%
Independent	Kd (M)	6,719E-7	±1,547E-7	95%
	n	1,043	±0,019	95%
	ΔH (kcal/mol)	-4,976	±0,123	95%
	-TΔS (kcal/mol)	-3,445		
	ΔG (kcal/mol)	-8,421		
	ΔS (cal/mol·K)	11,56		

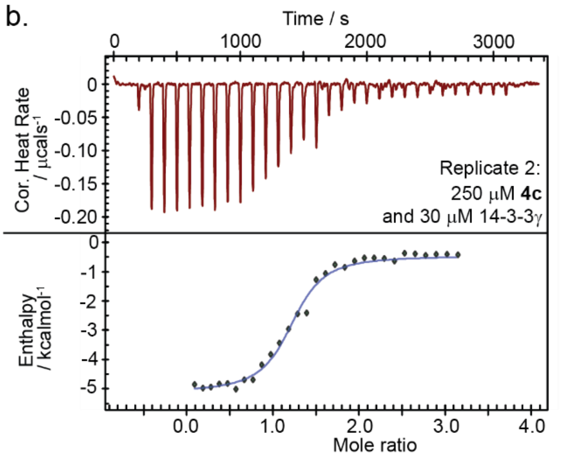

Model	Variable	Value	Confidence Interval	Confidence Level
Blank (constant)	blank (µcal)	-0,22991	±0,00000	95%
Independent	Kd (M)	8,558E-7	±2,746E-7	95%
	n	1,206	±0,033	95%
	ΔH (kcal/mol)	-4,649	±0,169	95%
	-TΔS (kcal/mol)	-3,629		
	ΔG (kcal/mol)	-8,278		
	ΔS (cal/mol·K)	12,17		

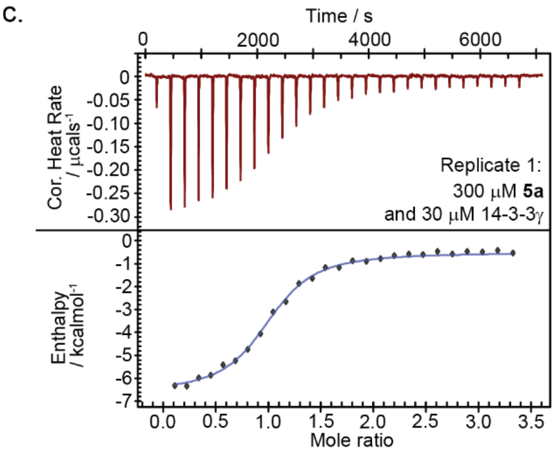

Model	Variable	Value	Confidence Interval	Confidence Level
Blank (constant)	blank (µcal)	-0,30958	±0,00000	95%
Independent	Kd (M)	1,553E-6	±2,669E-7	95%
	n	0,997	±0,021	95%
	ΔH (kcal/mol)	-6,091	±0,171	95%
	ΔS (cal/mol·K)	6,150		

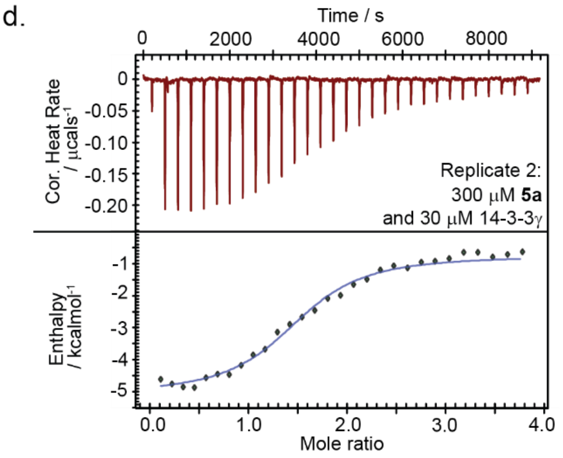

Model	Variable	Value	Confidence Interval	Confidence Level
Blank (constant)	blank (µcal)	-0,44484	±0,00000	95%
Independent	Kd (M)	2,570E-6	±7,194E-7	95%
	n	1,510	±0,053	95%
	ΔH (kcal/mol)	-4,328	±0,204	95%
	ΔS (cal/mol·K)	11,06		

Figure S10C. Raw ITC data 4c and 5a. Measured raw thermograms, binding isotherms and derived thermodynamic values for 14-3-3γ binding to **4c** (Nph+1Ala) and **5a** (Nph+4Tyr). Used concentrations are state in the graph. A constant blank model is used to correct for heat of injection and independent model is used to model peptide binding (blue line). From this the thermodynamic parameters are determined as noted in each table associated with the thermograms. Each peptide is measured in two independent experiments (replicate 1 and 2) which are both shown here. **For thermodynamic parameters refer to Table S6.

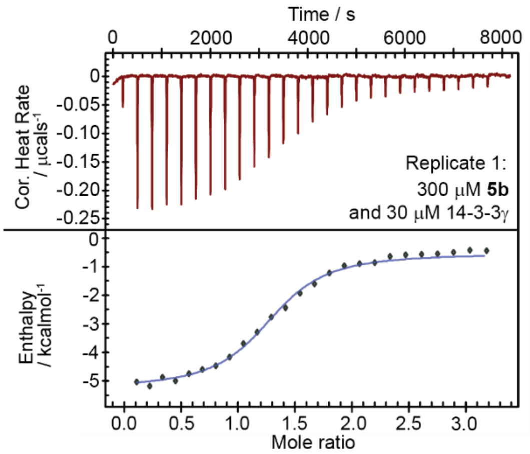

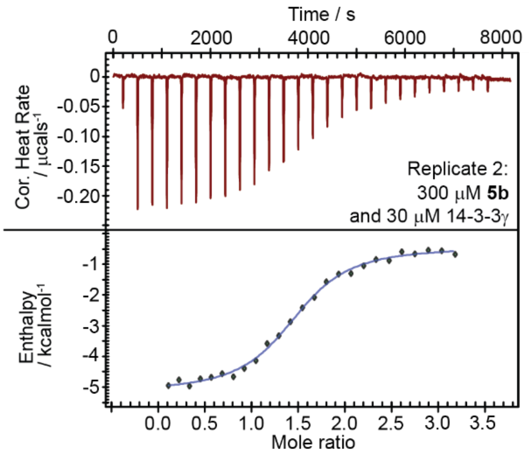

Model	Variable	Value	Confidence Interval	Confidence Level
Blank (constant)	blank (µcal)	-0,32068	±0,00000	95%
Independent	Kd (M)	1,506E-6	±3,292E-7	95%
	n	1,277	±0,030	95%
	ΔH (kcal/mol)	-4,716	±0,143	95%
	ΔS (cal/mol·K)	10,82		

Model	Variable	Value	Confidence Interval	Confidence Level
Blank (constant)	blank (µcal)	-0,25531	±0,07017	95%
Independent	Kd (M)	1,849E-6	±4,806E-7	95%
	n	1,470	±0,037	95%
	ΔH (kcal/mol)	-4,725	±0,228	95%
	ΔS (cal/mol·K)	10,38		

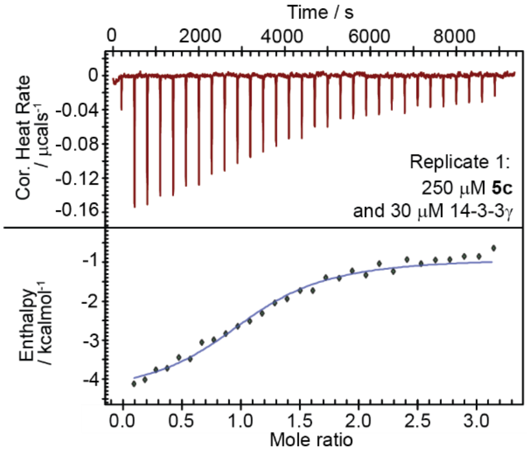

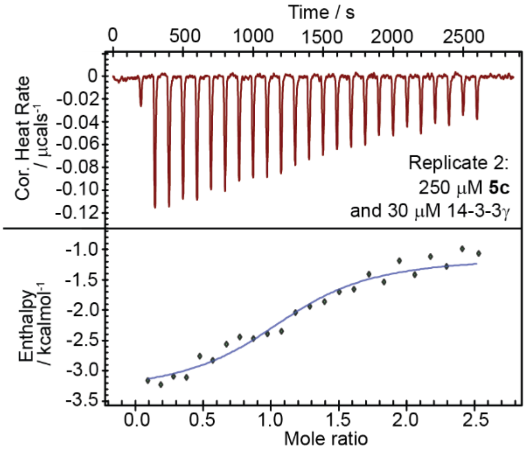

Model	Variable	Value	Confidence Interval	Confidence Level
Blank (constant)	blank (µcal)	-0,55564	±0,00000	95%
Independent	Kd (M)	3,482E-6	±1,970E-6	95%
	n	1,144	±0,092	95%
	ΔH (kcal/mol)	-2,242	±0,261	95%
	-TΔS (kcal/mol)	-5,204		
	ΔG (kcal/mol)	-7,446		
	ΔS (cal/mol·K)	17,45		

Model	Variable	Value	Confidence Interval	Confidence Level
Blank (constant)	blank (µcal)	-0,43617	±0,00000	95%
Independent	Kd (M)	4,328E-6	±1,467E-6	95%
	n	1,107	±0,065	95%
	ΔH (kcal/mol)	-3,539	±0,290	95%
	-TΔS (kcal/mol)	-3,779		
	ΔG (kcal/mol)	-7,317		
	ΔS (cal/mol·K)	12,67		

Figure S10D. Raw ITC data 5b and 5c. Measured raw thermograms, binding isotherms and derived thermodynamic values for 14-3-3γ binding to **5b** (Nph+4Phe) and **5c** (Nph+4Ala). Used concentrations are state in the graph. A constant blank model is used to correct for heat of injection and independent model is used to model peptide binding (blue line). From this the thermodynamic parameters are determined as noted in each table associated with the thermograms. Each peptide is measured in two independent experiments (replicate 1 and 2) which are both shown here.**For thermodynamic parameters refer to Table S6.

Table S5. Overview K_d and ΔΔG analysis mutated peptides (ITC-based assays). K_d values are determined from the ITC-based binding studies of 14-3-3 to each of the peptides. The fold change in binding affinity is determined by dividing the K_d from a peptide with the K_d of peptide **1**. Furthermore the K_d is transformed into ΔG values which in the end is transformed into a ΔΔG analysis by subtracting the ΔG from each peptide with the ΔG from peptide **1**.

Affinity Kd (M)	1	3a	4a	4b	4c	5a	5b	5c
Mutant	-	Nph+4Ala	Nph+1Tyr	Nph+1Phe	Nph+1Ala	Nph +4Tyr	Nph +4Phe	Nph +4Ala
Replicate 1	4.5E-08	8.9E-08	1.3E-07	2.6E-08	6.7E-07	1.6E-06	1.1E-06	3.5E-06
Replicate 2	4.5E-08	7.0E-08	6.1E-08	3.8E-08	8.6E-07	2.6E-06	1.8E-06	4.3E-06
Mean	4.5E-08	7.9E-08	9.4E-08	3.2E-08	7.6E-07	2.1E-06	1.5E-06	3.9E-06
Std. deviation	4.2E-10	1.3E-08	4.6E-08	8.1E-09	1.3E-07	7.2E-07	5.6E-07	6.0E-07

Fold change to 1	1	3a	4a	4b	4c	5a	5b	5c
Mutant	-	Nph+4Ala	Nph+1Tyr	Nph+1Phe	Nph+1Ala	Nph +4Tyr	Nph +4Phe	Nph +4Ala
		2.0	2.8	0.6	15.0	15.0	34.7	23.6
		1.5	1.3	0.8	18.9	18.9	56.7	40.8
Mean		1.8	2.1	0.7	17.0	17.0	45.7	32.2
		0.3	1.0	0.2	2.7	2.7	15.6	12.2

ΔG (kcal/mol)	1	3a	4a	4b	4c	5a	5b	5c
Mutant	-	Nph+4Ala	Nph+1Tyr	Nph+1Phe	Nph+1Ala	Nph +4Tyr	Nph +4Phe	Nph +4Ala
Replicate 1	-10.0	-9.6	-9.4	-10.3	-8.4	-7.9	-7.9	-7.4
Replicate 2	-10.0	-9.8	-9.8	-10.1	-8.3	-7.6	-7.8	-7.3
Mean	-10.0	-9.7	-9.6	-10.2	-8.3	-7.8	-7.9	-7.4
Std. deviation	0.0	0.1	0.3	0.1	0.1	0.2	0.1	0.1

ΔΔG (kcal/mol) to 1	1	3a	4a	4b	4c	5a	5b	5c
Mutant	-	Nph+4Ala	Nph+1Tyr	Nph+1Phe	Nph+1Ala	Nph +4Tyr	Nph +4Phe	Nph +4Ala
		0.4	0.6	-0.3	1.6	2.1	2.1	2.6
		0.3	0.2	-0.1	1.7	2.4	2.2	2.7
Mean		0.3	0.4	-0.2	1.7	2.2	2.1	2.6
Std. deviation		0.1	0.3	0.1	0.1	0.2	0.1	0.1

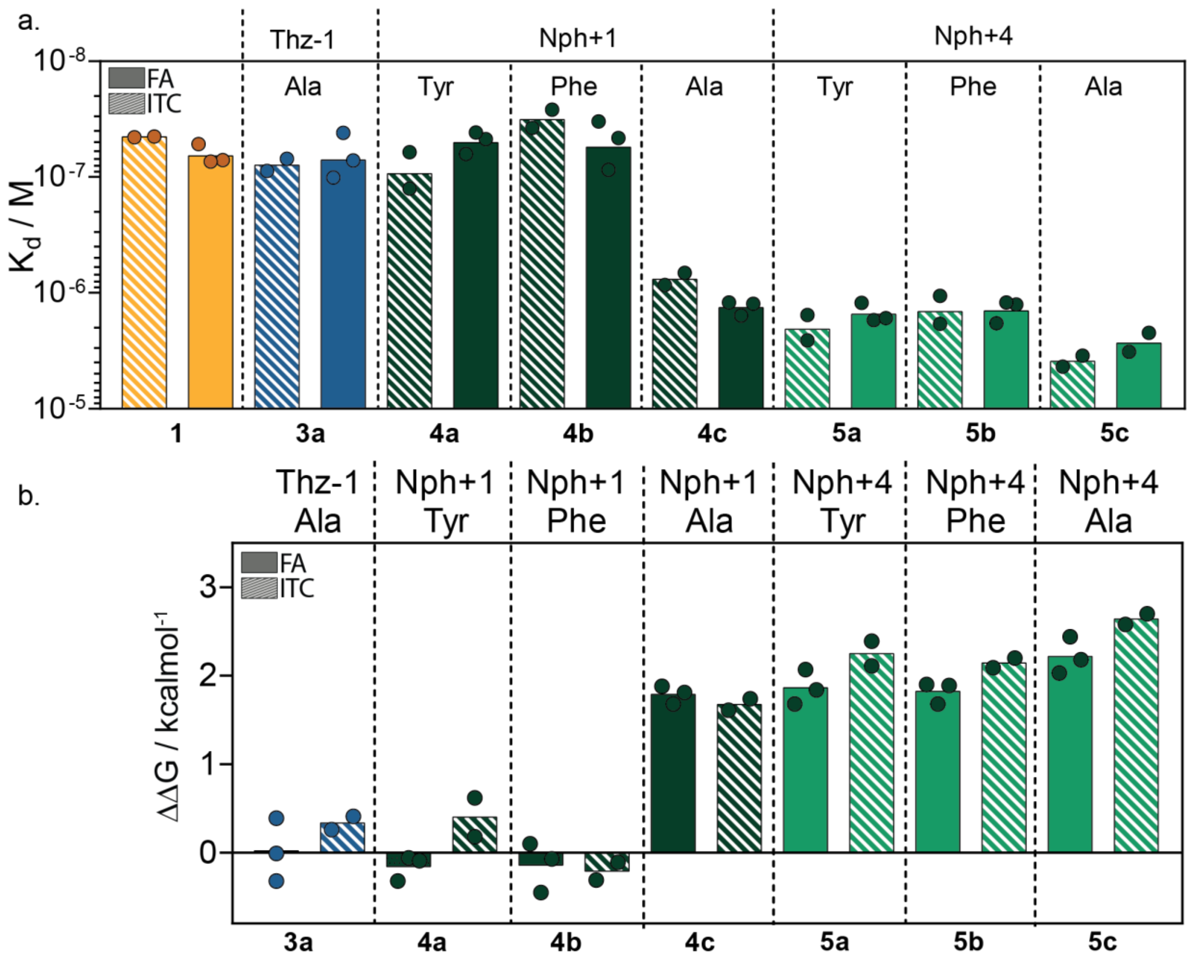

Figure S11. K_D and ΔΔG analysis of mutant peptides. (a) Bar plot representation of K_D values obtained from both ITC and FP experiments for peptide **1** and the Thz-1 (**3a**), Nph+1 (**4a-c**), and Nph+4 (**5a-c**) mutants. (b) Bar plot representation of the DDG contributions of each Thz-1, Nph+1 and Nph+4 Thz-1 (**3a**), Nph+1 (**4a-c**), and Nph+4 (**5a-c**) mutation as obtained in both ITS and FP experiments.

Table S6. Thermodynamic parameters ITC mutated peptides. Obtained K_d , ΔH, -TΔS, and ΔG values for **1** and point mutated peptides. Two runs were performed for each peptide and for each of the parameters the average and standard deviation has been calculated.

**Run 1**	**1**	**3a**	**4a**	**4b**	**4c**	**5a**	**5b**	**5c**
	**-**	**Nph+4Ala**	**Nph+1Tyr**	**Nph+1Phe**	**Nph+1Ala**	**Nph +4Tyr**	**Nph +4Phe**	**Nph +4Ala**
**Kd**	4,47E-08	8,86E-08	1,26E-07	2,62E-08	6,72E-07	1,55E-06	1,06E-06	3,48E-06
**ΔH**	-10,96	-7,998	-5,83	-8,024	-4,976	-6,091	-4,716	-2,242
**−TΔS**	0,933	-1,624	-3,582	-2,391	-3,445	-1,834	-3,227	-5,204
**ΔG**	-10,03	-9,622	-9,411	-10,34	-8,421	-7,925	-7,943	-7,446

**Run 2**	**1**	**3a**	**4a**	**4b**	**4c**	**5a**	**5b**	**5c**
	**-**	**Nph+4Ala**	**Nph+1Tyr**	**Nph+1Phe**	**Nph+1Ala**	**Nph +4Tyr**	**Nph +4Phe**	**Nph +4Ala**
**Kd**	4,53E-08	6,97E-08	6,09E-08	3,76E-08	8,56E-07	2,57E-06	1,85E-06	4,33E-06
**ΔH**	-10,45	-6,809	-7,45	-6,783	-4,649	-4,328	-4,725	-3,539
**−TΔS**	0,426	-2,955	-2,393	-3,345	-3,629	-3,299	-3,096	-3,779
**ΔG**	-10,02	-9,764	-9,844	-10,13	-8,278	-7,626	-7,821	-7,317

**Average**	**1**	**3a**	**4a**	**4b**	**4c**	**5a**	**5b**	**5c**
	**-**	**Nph+4Ala**	**Nph+1Tyr**	**Nph+1Phe**	**Nph+1Ala**	**Nph +4Tyr**	**Nph +4Phe**	**Nph +4Ala**
**Kd**	4,50E-08	7,91E-08	9,36E-08	3,19E-08	7,64E-07	2,06E-06	1,45E-06	3,91E-06
**ΔH**	-10,71	-7,40	-6,64	-7,40	-4,81	-5,21	-4,72	-2,89
**−TΔS**	0,68	-2,29	-2,99	-2,87	-3,54	-2,57	-3,16	-4,49
**ΔG**	-10,03	-9,69	-9,63	-10,24	-8,35	-7,78	-7,88	-7,38

**Std. deviation**	**1**	**3a**	**4a**	**4b**	**4c**	**5a**	**5b**	**5c**
	**-**	**Nph+4Ala**	**Nph+1Tyr**	**Nph+1Phe**	**Nph+1Ala**	**Nph +4Tyr**	**Nph +4Phe**	**Nph +4Ala**
**Kd**	4,17E-10	1,34E-08	4,62E-08	8,08E-09	1,30E-07	7,19E-07	5,61E-07	5,98E-07
**ΔH**	0,36	0,84	1,15	0,88	0,23	1,25	0,01	0,92
**−TΔS**	0,36	0,94	0,84	0,67	0,13	1,04	0,09	1,01
**ΔG**	0,01	0,10	0,31	0,15	0,10	0,21	0,09	0,09

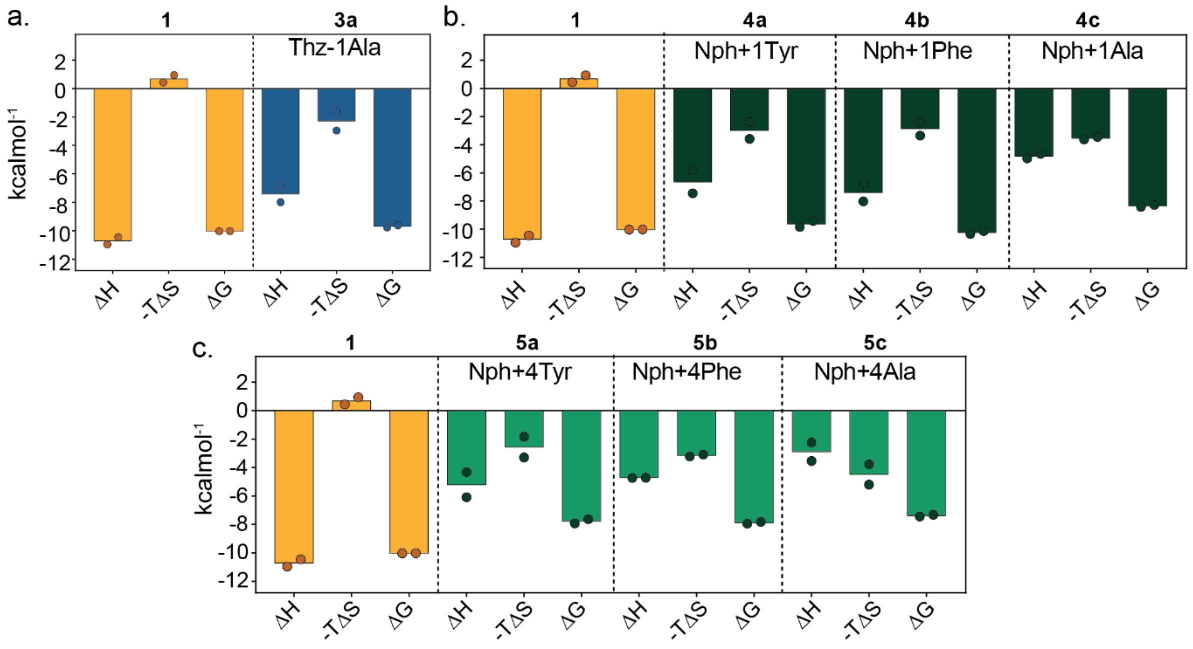

Figure S12. ITC thermodynamic parameters. Bar plot representation of thermodynamic parameters as obtained from ITC. For each peptide the thermodynamic binding characteristics ΔH, -TΔS and ΔG, are shown for its binding to 14-3-3g. (a) **1** and **3a** (Thz-1Ala). (b) **1** and **4a-c** (Nph+1 mutants). (c) **1** and **5a-c** (Nph+4 mutants).

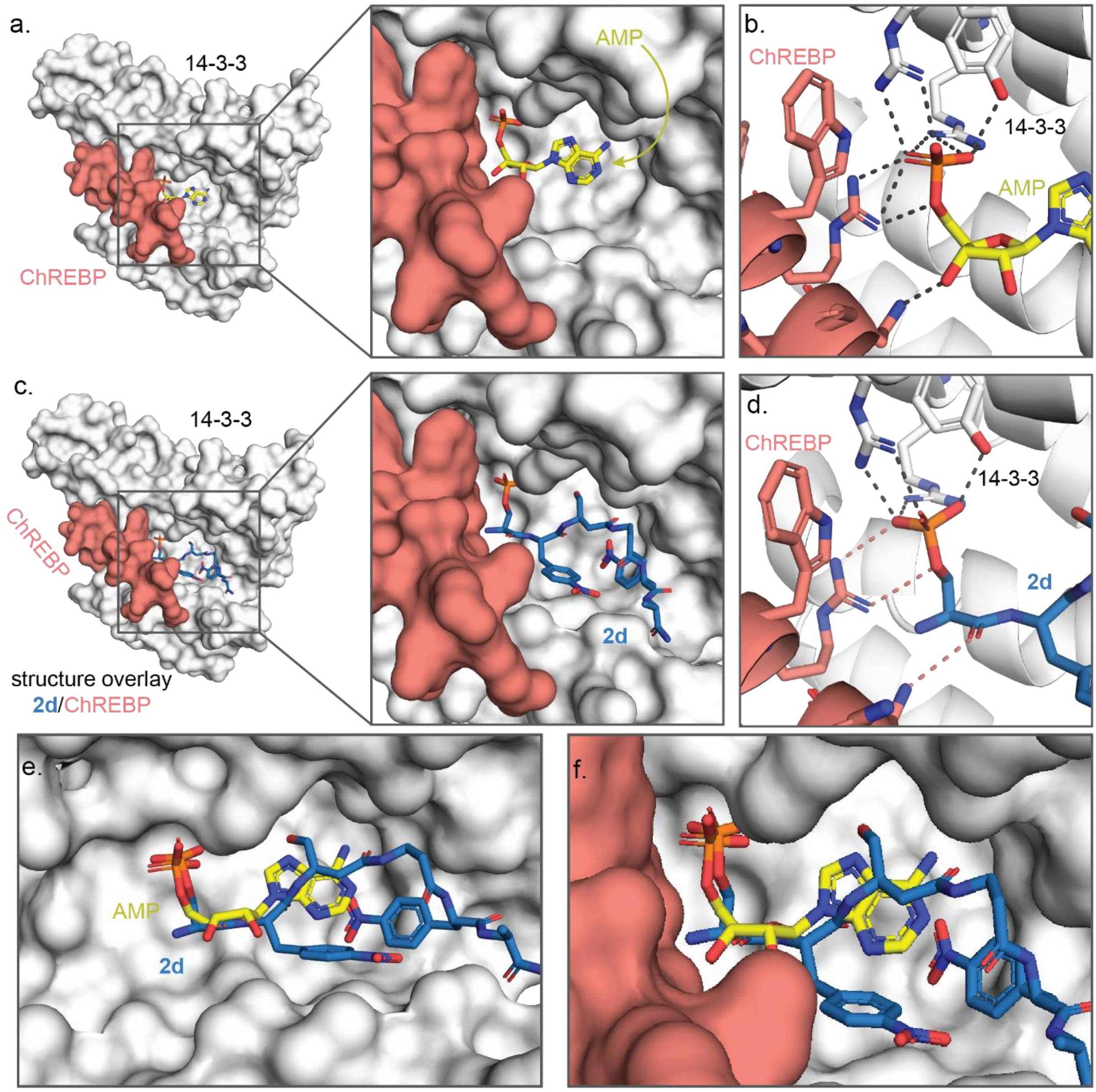

Figure S13. Overlay 14-3-3 binding to AMP/ChREBP and to peptide 2d. (a) Crystal structure of 14-3-3 (white surface) bound to ChREBP (salmon surface) and AMP (yellow sticks) binding as a stabilizer at the interface between 14-3-3 and ChREBP. PDB: 5F74 (b) Hydrogen bonding and electrostatic interactions (black dashes) between AMP and both 14-3-3 and ChREBP highlighting the binding of AMP on the PPI interface. (c) Structural overlay of 14-3-3/ChREBP and the 14-3-3/**2d (blue sticks)** binary complexes showing complementarity of 2d anc ChREBP binding to 14-3-3. (d) Enlarged view of phosphate group of peptide **2d** interacting with the phosphoaccepting group of 14-3-3 (black dashes) and its potential interactions with ChREBP (salmon dashes). Showing the potential of **2d** to act as a cooperative ligand between 14-3-3 and ChREBP. (e-f) Structural overlay of AMP and **2d** binding to 14-3-3 (and ChREBP) showing similarity in binding of both AMP and **2d** in the phosphate binding pocket.

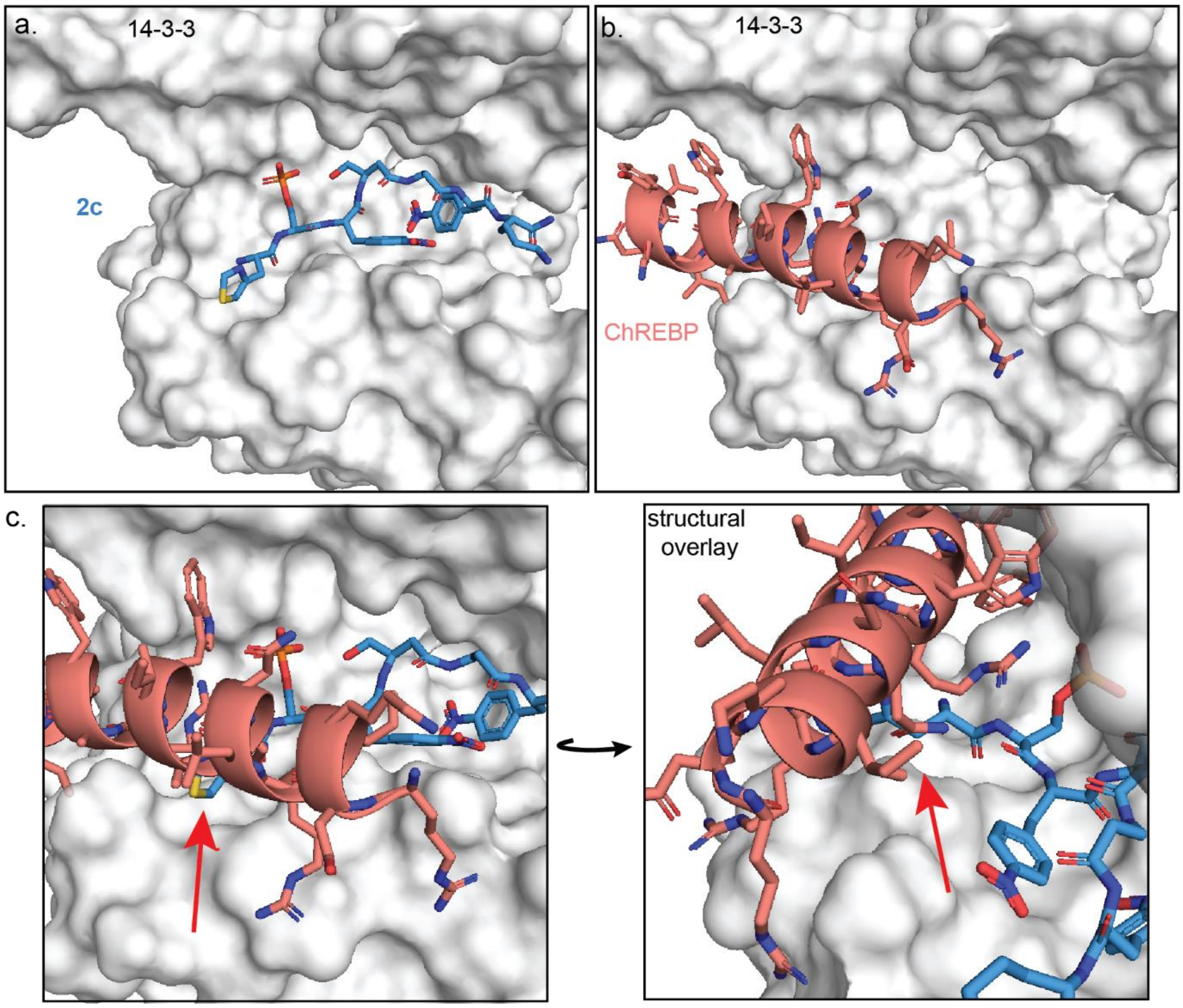

Figure S14. Overlay ChREBP and peptide 2c. (a) Crystal structure of 14-3-3 (white surface) bound to **2c** (blue sticks), PDB: 7ZMW. (b) Crystal structure of 14-3-3 (white surface) bound to ChREBP peptide (salmon sticks and cartoon), PDB: 5F74. (c) Structural overlay of **2c** with thiazolylalanine (Thz) at -1 position (blue sticks) and ChREBP (salmon sticks/cartoon) bound to 14-3-3 (white surface) showing a clear steric clash (red arrow) in peptide binding.

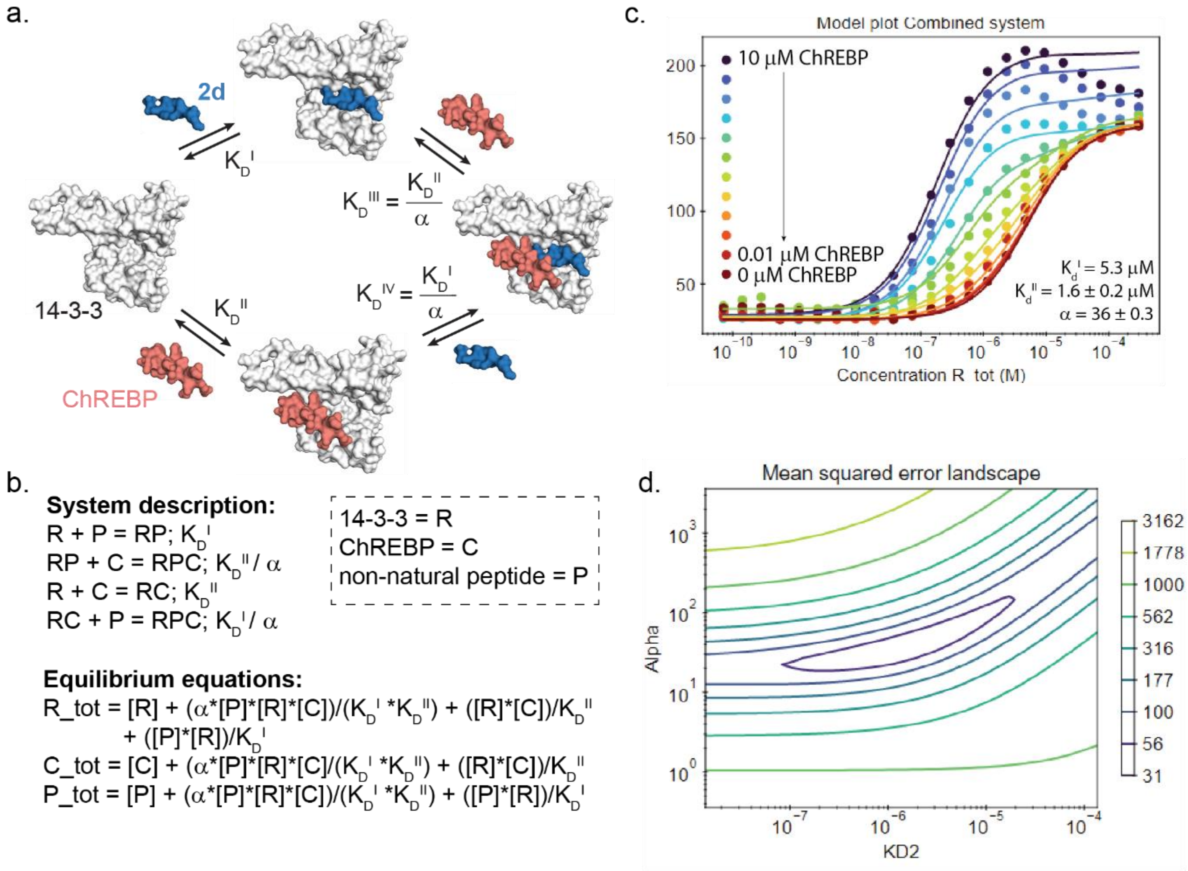

Figure S15. Cooperativity analysis. (a) Cooperativity square representation of the 14-3-3/ChREBP/**2d** complex formation including binding affinities. The interaction is stabilized by factor alpha (α), reducing the apparent affinity of 14-3-3 to either of the components when pre-bound to the other component. (b) System description given to model to determine our ternary complex formation system and the determined equilibrium equations as generated by the model. (c) Experimental fluorescence anisotropy data of 14-3-3 titration to FITC-labelled **2d** in presence of several concentrations ChREBP peptide. All the given K_D^I and calculated K_D^{II} and a factor are given with a 95% confidence interval. (d) Error-landscape plot centered on the determined K_D^{II} and α factors. The contours show that there is a valley of parameter combinations that result in a relatively low mean squared error (MSE).

Table S7. Overview peptides used in selectivity studies including the phosphorylation site, peptide sequence, PDB reference for crystal structure and reference.

Protein	Phosphosite	Sequence	PDB	Ref.
ChREBP	-	Ac-DKIRLNNAIWRAWYIQYVKRRKSPV-$CONH_2$	5F74	1
ERa	pT594	Ac-AEGFPA pT V-COOH	4JC3	2,3
SOS1	pS1161	Ac-PRRRPE pS APAES-$CONH_2$	6F08	4
BRAF	pS365	Ac-RDRSS pS APNVH-$CONH_2$	-	5
ERRg	pS79	Ac-KRRRK pS CQA-$CONH_2$	6Y1D	6
USP8	pS718	Ac-KLKRSY pS SPDITQ-$CONH_2$	6F09	7
PIN1	pS72	Ac- LVKHSQSRRPS pS WRQEK-$CONH_2$	7AOG	8
P65	pS45	Ac- EGRSAG pS IPGRRS-$CONH_2$	6YOW	9

Table S8. Data collection and refinement statistics (molecular replacement) for 14-3-3σΔc in complex with non-natural N-terminally truncated peptides **2c** and **2d**

**14-3-3σΔc in complex with N-terminal truncated peptides 2c-d**		
PDB	7ZMU	7ZMW
Peptide	**2d**	**2c**
Beam	DESY p11	Homesource
Data collection		
Space group	C 2 2 21	C 2 2 21
Cell dimensions<br>a, b, c (Å)<br>α, β, γ (°)	 82.3 112.0 62.7<br>90, 90, 90	 82.0 111.6 62.5<br>90, 90, 90
Resolution (Å)	45.55 – 1.60 (1.63 – 1.60)	33.02 – 1.80 (1.84-1.80)
$I / \sigma(I)$	8.2 (3.5)	11.2 (3.0)
Completeness (%)	100.0 (99.9)	97.4 (90.1)
Redundancy	13.4 (12.3)	6.0 (5.0)
$CC_{1/2}$	0.988 (0.917)	0.996 (0.893)
Refinement		
No. reflections	38533	26185
R_{work}/R_{free}	0.253/0.294	0.179/0.221
No. atoms<br>Protein<br>Ligand/ion<br>Water	 1975<br>112<br>253	 1871<br>127<br>267
B-factors<br>Protein<br>Ligand/ion<br>Water	 16.22<br>17.61<br>25.98	 12.48<br>26.05<br>22.17
R.m.s. deviations<br>Bond lengths (Å)<br>Bond angles (°)	 0.012<br>1.24	 0.011<br>1.26
Ramachandran<br>favored (%)<br>outliers (%)	 98.29<br>0.00	 97.86<br>0.00

Experimental procedures

14-3-3γ protein expression (FP/ITC experiments)

A pPROEX HTb expression vector encoding the human 14-3-3 protein gamma (14-3-3γ) with an N-terminal his_6-tag was transformed by heat shock into NiCo21 (DE3) competent cells. Single colonies were cultured in 50 mL LB medium (100 μg/mL ampicillin). After overnight incubation at 37 °C, cultures were transferred to 2 L TB media (100 μg/mL ampicillin, 1 mM $MgCl_2$) and incubated at 37 °C until an OD600 nm of 0.8-1.2 was reached. Protein expression was then induced with 0.4 mM isopropyl-β-d-thiogalactoside (IPTG), and cultures were incubated overnight at 18˚C. Cells were harvested by centrifugation (8600 rpm, 20 minutes, 4˚C) and resuspended in lysis buffer (50 mM Hepes, pH 8.0, 300 mM NaCl, 12.5 mM imidazole, 5 mM $MgCl_2$, 2 mM βME) containing cOmplete™ EDTA-free Protease Inhibitor Cocktail tablets (1 tablet/100 ml lysate) and benzonase (1 μl/100 ml). After lysis using a C3 Emulsiflex-C3 homogenizer (Avestin), the cell lysate was cleared by centrifugation (20000 rpm, 30 minutes, 4˚C) and purified using Ni^{2+}-affinity chromatography (Ni-NTA superflow cartridges, Qiagen). Typically two 5 mL columns (flow 5 mL/min) were used for a 2 L culture in which the lysate was loaded on the column washed with 10 CV wash buffer (50 mM Hepes, pH 8.0, 300 mM NaCl, 25 mM imidazole, 2 mM βME) and eluted in several fractions (2-4 CV) of elution buffer (50 mM Hepes, pH 8.0, 300 mM NaCl, 250 mM imidazole, 2 mM βME). Fractions containing the 14-3-3 protein were combined and dialyzed into 25 mM HEPES pH 8.0, 100 mM NaCl, 10 mM $MgCl_2$, 500 μM TCEP. Finally, the protein was concentrated to ~60 mg/mL, analyzed Q-Tof LC/MS, and aliquots were flash-frozen for storage at −80 °C.

14-3-3σΔc protein expression (Crystallography)

A pPROEX HTb expression vector encoding the human 14-3-3 protein sigma truncated after T231 (14-3-3σΔc) and with an N-terminal his6-tag was transformed by heat shock into NiCo21 (DE3) competent cells. Single colonies were cultured in 50 mL LB medium (100 μg/mL ampicillin). After overnight incubation at 37 °C, cultures were transferred to 2 L TB media (100 μg/mL ampicillin, 1 mM MgCl2) and incubated at 37 °C until an OD600 nm of 0.8-1.2 was reached. Protein expression was then induced with 0.4 mM isopropyl-β-d-thiogalactoside (IPTG), and cultures were incubated overnight at 18˚C. Cells were harvested by centrifugation (8600 rpm, 20 minutes, 4 ˚C) and resuspended in lysis buffer (50 mM Hepes, pH 8.0, 300 mM NaCl, 12.5 mM imidazole, 5 mM $MgCl_2$, 2 mM βME) containing cOmplete™ EDTA-free Protease Inhibitor Cocktail tablets (1 tablet/ 100 ml lysate) and benzonase (1 μl/ 100 ml). After lysis using a C3 Emulsiflex-C3 homogenizer (Avestin), the cell lysate was cleared by centrifugation (20000 rpm, 30 minutes, 4 ˚C) and purified using Ni2+-affinity chromatography (Ni-NTA superflow cartridges, Qiagen). Typically two 5 mL columns were used for a 2 L culture in which the lysate was loaded on the column washed with 10 CV wash buffer (50 mM Hepes, pH 8.0, 300 mM NaCl, 25 mM imidazole, 2 mM βME) and eluted with several fractions (2-4 CV) of elution buffer (50 mM Hepes, pH 8.0, 300 mM NaCl, 250 mM imidazole, 2 mM βME). Fractions containing the 14-3-3 protein were combined and dialyzed into 25 mM HEPES pH 8.0, 200 mM NaCl, 10 mM MgCl2, 2 mM βME. In addition, 1 mg TEV was added for each 100 mg purified protein to remove the purification tag. The cleaved sample was then again loaded on a 10 mL Ni-NTA column to separate the cleaved product from the expression tag and residual uncleaved protein. The flowthrough was loaded on a Superdex 75 pg 16/60 size exclusion column (GE Life Sciences) using 25 mM HEPES, 100 mM NaCl, 10 mM $MgCl_2$, 500 μM TCEP (adjusted to pH=8.0) as running buffer. Fractions containing the 14-3-3 protein were pooled and concentrated to ~60 mg/mL, analyzed Q-Tof LC/MS, and aliquots were flash-frozen for storage at −80 °C.

Peptide synthesis

The peptides **1** and **2a-d** were synthesized on 50 μmol scale on a Rink amide MBHA resin (Novabiochem, 0.52 mmol/g loading) using an automated Intavis MultiPep RSi peptide synthesizer. Deprotection was performed twice per cycle with 20% v/v piperidine in DMF for 8 minutes. Fmoc-protected amino acids were mixed in 5 equivalents of HBTU and 9 equivalents of DIPEA in DMF, and coupled to the resin. With the exception of phosphoserine, which was coupled once for 60 minutes, all amino acids were coupled to the resin twice for 30 minutes. Capping of unreacted amino acids was performed with a mixture of acetic anhydride/pyridine/DMF (1:1:3) for 5 minutes. βAla was incorporated as a spacer between the sequence and Lys(Alloc), to which a FITC fluorophore will be coupled for fluorescence anisotropy studies.

The peptides **1** and **2e-i** were synthesized manually on a 50 μmol scale using Rink amide MBHA resin (Novabiochem, 0.52 mmol/g loading) via Fmoc based solid phase peptide synthesis. Deprotection was

performed twice per cycle with 20% v/v piperidine in N,N-dimethylformamide (DMF) for five minutes. Fmoc-protected amino acids were mixed with 9 equivalents HBTU (0.38 M in DMF) and 15 equivalents of DIPEA, and coupled to the resin for 30 minutes (except pS, which is coupled for 60 minutes).
Peptides **1** and **2e-i** were labeled with fluorescein via a 5-(-fluorenylmehyloxycarbonyl-amino)-3-oxapentanoic acid (O1-Pen) linker using 5 equivalents of FITC and 7.5 equivalents of DIPEA reacting overnight with continuous agitation. Removal of protecting groups and cleavage of the resin was performed by incubation in a mixture of trifluoroacetic acid (TFA), H_2O, and triisopropylsilane (TIS) (96.5:2.5:1) for 2 hours with continuous agitation, followed by precipitation in an excess of ice-cold diethyl ether (Et_2O).
Peptides **1** and **2a-d** were FITC labeled via a three-step approach. The free amine on the N-terminus was protected as follows: to a solution of di-tert-butyl decarbonate (10 eq, 400 mM) in DCM was added DIEA (10 eq), and solution was added to the resin. Coupling was allowed to proceed for 1 h. At this time, resin was washed 3x with DCM and coupling was repeated as described. Resin was washed 5x with DCM. Alloc removal was achieved as follows: resin was treated with a solution of tetrakis(triphenylphosphine)palladium(0) (0.5 eq, 20 mM) and phenylsilane (20 eq) in DCM, 2x 45 min. Resins were then washed 3x with DCM, then 3x with DMF. FITC was installed on the free amine on each C-terminal lysine by treating resin with fluorescein isothiocyanate isomer I (10 eq, 400 mM in 4:1 DMF:DCM) and DIEA (15 eq) for at least 1.5 h. Reactions were kept under aluminum foil for the duration of the coupling. Reaction mixtures were then drained and resins were washed 3x with DMF, 3x with DCM, and dried under reduced pressure. Removal of protecting groups and cleavage of the resin was performed by incubation in a mixture of trifluoroacetic acid (TFA), H_2O, and triisopropylsilane (TIS) (96.5:2.5:1) for 2 hours with continuous agitation, followed by precipitation in excess of ice-cold diethyl ether (Et_2O).

All peptides were purified using preparative HP-LC. This was performed using a Gemini S4 110A 150 x 21.20 mm column using ultrapure water with 0.1% formic acid (FA) and acetonitrile with 0.1% FA with various gradients. Correct mass and purity of peptides was identified using analytical liquid-chromatography coupled with mass-spectrometry (LC-MS) was performed on a C4 Jupiter SuC4300A 150 x 2.0 mm column using ultrapure water with 0.1% formic acid (FA) and acetonitrile with 0.1% FA, in general with a gradient of 5% to 100% acetonitrile over 10 minutes, connected to a Thermo Fisher LCQ Fleet Ion Trap Mass Spectrometer. The purity of the samples was assessed using a UV detector at 254 nm.

Fluorescence anisotropy (FA) - binary binding studies: 14-3-3γ was titrated in a 2-fold dilution series (starting at 100 or 400 μM 14-3-3γ) to 10 nM of fluorescein-labeled peptide in FA buffer (10 mM HEPES pH 7.4, 150 mM NaCl, 0.1% (v/v) Tween20, 0.1% (w/v) BSA). Dilution series were made in a polystyrene (non-binding) low-volume Corning Black Round Bottom 384-well plates (Corning 4514 or 4511). Measurements were performed directly after plate preparation, using a Tecan Infinite F500 plate reader at room temperature (l_{ex}: 485 ± 20 nm; l_{em}: 535 ± 25 nm; mirror: Dichroic 510; flashes: 20; integration time: 50 ms; settle time: 0 ms; gain: optimal; and Z-position: calculated from well). Wells containing only FITC-peptide were used to set as G-factor at 35 mP. All data were analyzed using GraphPad Prism (7.00) for Windows and fitted using a four-parameter logistic model (4PL). Each measurement was performed in three independent experiments, average and standard deviations were calculated in Excel (see SI tables S1-S4).

ΔΔG analysis: Based on the obtained binding affinities from the FA binding studies the fold change in binding affinity is determined by dividing the K_D of one peptide with the K_D from another peptide (fold change = $K_D^{peptide1}$/ $K_D^{peptide2}$). Furthermore the K_D can be change into a ΔG (in kcal/mol) value using ΔG = (ln(K_D)*-8,314*298)/4184. Final ΔΔG value is obtained by subtracting ΔG values from one peptide with the ΔG value of another peptide (see SI tables S2-S4).

Fluorescence anisotropy (FA) - ChREBP titrations: ChREBP peptide was titrated in a 2-fold dilution series (starting at 100 μM) to 10 nM of fluorescein-labeled peptide **2c** and **2d** in presence and absence of, respectively, 2 μM and 40 nM 14-3-3γ in FA buffer (10 mM HEPES pH 7.4, 150 mM NaCl, 0.1% (v/v) Tween20, 0.1% (w/v) BSA). Dilution series were made in a polystyrene (non-binding) low-volume Corning Black Round Bottom 384-well plates (Corning 4514 or 4511). Measurements were performed directly after plate preparation, using a Tecan Infinite F500 plate reader at room temperature (l_{ex}: 485 ± 20 nm; l_{em}: 535 ± 25 nm; mirror: Dichroic 510; flashes: 20; integration time: 50 ms; settle time: 0 ms; gain: optimal; and Z-position: calculated from well). Wells containing only FITC-peptide were used to set as G-factor at 35 mP. All data were analyzed using GraphPad Prism (7.00) for Windows and fitted using

a four-parameter logistic model (4PL). Each measurement was performed in two independent experiments.

Fluorescence anisotropy (FA) - 2D-14-3-3/ChREBP titrations: 14-3-3γ was titrated in a 2-fold dilution series (starting at 300 μM 14-3-3γ) to 20 nM of fluorescein-labeled peptide **2d** in FA buffer (10 mM HEPES pH 7.4, 150 mM NaCl, 0.1% (v/v) Tween20, 0.1% (w/v) BSA) in presence of 2-fold dilution series of unlabeled ChREBP peptide (0.01-10 μM). Dilution series were made in a polystyrene (non-binding) low-volume Corning Black Round Bottom 384-well plates (Corning 4514 or 4511). Measurements were performed directly after plate preparation, using a Tecan Infinite F500 plate reader at room temperature (l_{ex}: 485 ± 20 nm; l_{em}: 535 ± 25 nm; mirror: Dichroic 510; flashes: 20; integration time: 50 ms; settle time: 0 ms; gain: optimal; and Z-position: calculated from well). Wells containing only FITC-peptide were used to set as G-factor at 35 mP. All data were analyzed using GraphPad Prism (7.00) for Windows and fitted using a four-parameter logistic model (4PL). Data was obtained and averaged based on two independent experiments.

Cooperativity analysis:
To determine the cooperativity parameters from the 2D-titration of 14-3-3/ChREBP/**2d** (as described above) we have used the general framework for straightforward model construction of multi-component thermodynamic equilibrium systems as described by Geertjens et al. (2021).[10] This general platform generates a model to describe multi-component equilibrium systems when given a system description. In our case we gave the following system description:

$R + P = RP; K_D^I$
$RP + C = RPC; K_D^{II} / \alpha$
$R + C = RC; K_D^{II}$
$RC + P = RPC; K_D^I / \alpha$

with R = 14-3-3, P = non-natural peptide (labelled), and C = ChREBP. The framework determined based on this system description the equilibrium equations needed to determine K_D^{II} and α. The data from 2D-titrations was provided to the model including the K_D^I at 1.96 mM, P_tot = 10 nM, and the variable concentrations of 14-3-3 and ChREBP at each datapoint. From this the model determined the K_D^{II} and α (with 95% confidence interval) and the error-landscape of the determine parameters.

Fluorescence anisotropy (FA) – Selectivity study: Peptides representing 14-3-3 binding partners (including ChREBP) were titrated in a 2-fold dilution series (starting at 100 μM) to 20 nM of fluorescein-labeled peptide **2d** in presence and absence of, respectively, 2 μM 14-3-3γ in FA buffer (10 mM HEPES pH 7.4, 150 mM NaCl, 0.1% (v/v) Tween20, 0.1% (w/v) BSA). Dilution series were made in a polystyrene (non-binding) low-volume Corning Black Round Bottom 384-well plates (Corning 4514 or 4511). Measurements were performed directly after plate preparation, using a Tecan Infinite F500 plate reader at room temperature (l_{ex}: 485 ± 20 nm; l_{em}: 535 ± 25 nm; mirror: Dichroic 510; flashes: 20; integration time: 50 ms; settle time: 0 ms; gain: optimal; and Z-position: calculated from well). Wells containing only FITC-peptide were used to set as G-factor at 35 mP. All data were analyzed using GraphPad Prism (7.00) for Windows and fitted using a four-parameter logistic model (4PL). Data was obtained and averaged based on two independent experiments.

Isothermal titration calorimetry (ITC)
Final dialysis fluid from protein expression was frozen as 2 mL aliquots to serve as ITC buffer. Protein and peptide were dissolved and diluted in this buffer to reported concentration. DMSO was added if reported and matched in cell and syringe. Samples were degassed for 10 min prior to measurement at 450 mmHg. The reference cell was filled with 300 μL degassed MilliQ water and sample cell with 300 μL of peptide or protein mixture. Syringe was loaded with at least 200 μL protein or peptide sample. Measurements were performed on an Affinity ITC LV (TA instruments), with injection size set to 2 μL, stirring speed of 150 rpm and temperature at 25 °C. The data was processed and analyzed in NanoAnalyze v3.11. The baseline was manually inspected and corrected, after which a blank constant model was fitted to correct for the heat of injection. Subsequently an independent model was fitted, which the Nanoanalyse uses to report the thermodynamic binding properties reported in this paper.

Crystallography
14-3-3σΔC/**2c**: Peptide **2c** was soaked in preformed crystals of 14-3-3σΔc (truncated after T231 to reduce flexibility) with cJun peptide[11], which grew in 28% (v/v) PEG400, 5% glycerol, 0.2 M $CaCl_2$, 0.1 M HEPES pH 7.5 within two weeks. The soaked crystal was fished after 14 days of incubation and flash-frozen in liquid nitrogen. Diffraction data was in-house collected at 100 K. X-ray diffraction data were

collected on an in-house Rigaku Micromax-003 (Rigaku, Europe, Kemsing Sevenoaks, UK) equiped with an Dectris Pilatus 200K detector, with the following settings: 360 image, 0.5°/image, 20 s exposure time.
14-3-3σΔC/**2d**: Peptide **2d** and 14-3-3 σΔC protein were mixed in complexation buffer (25 mM HEPES pH 7.5, 100 mM $MgCl_2$, and 2 mM βME) using a final 14-3-3 concentration of 12 mg/mL and a 1:4 protein-peptide molar ratio. After 1h complexation at room temperature, sitting drop crystallization wells were set-up using 250 nL complex mixture and 250 nL precipitation buffer (95 mM HEPES pH 7.1, 0.19 M $CaCl_2$, 5% glycerol and 27% PEG400). Crystals grew within 14 days at 4 °C. Suitable crystals were fished and flash-cooled in liquid nitrogen. X-ray diffraction data were collected at the p11 beamline of PETRA III facility at DESY (Hamburg, Germany) with the following settings: 1440 image, 0.25°/image, 100% transmission and 0.1 s exposure time. Initial data processing was performed at DESY using XDS after which pre-processed data was taken towards further scaling steps, molecular replacement and refinement.

Data was processed using the CCP4i2 suite (version 7.1.18).[12] DIALS[13] was used to index and integrate the data after which scaling was done using AIMLESS.[14,15] The data was phased with MolRep[16], using protein data bank (PDB) entry 4JC3 as a template. A three dimensional structure of peptide was generated using AceDRG[17], which was thereafter build in based on visual inspection Fo-Fc and 2Fo-Fc electron density map. Sequential model building (based on visual inspection Fo-Fc and 2Fo-Fc electron density map) and refinement were performed with COOT[18] and REFMAC[19-20], respectively. Finally, alternating cycles of model improvement (based on isotropic b-factors and standard set of stereo-chemical restraints: covalent bonds, angels, dihedrals, planarities, chiralities, non-bonded) and refinements were performed using coot and phenix.refine from the Phenix software suite (version 1.20.1-4487).[21-22] Pymol (version 2.2.3)[23] was used to make the figures and the structures were deposited in the protein data bank (PDB) with IDs: 7ZMU and 7ZMW.
See SI table S8 for crystal statistics.

References:

1. Sijbesma, E.; Visser, E.; Plitzko, K.; Thiel, P.; Milroy, L.; Kaiser, M.; Brunsveld, L.; Ottmann, C. Structure-Based Evolution of a Promiscuous Inhibitor to a Selective Stabilizer of Protein-Protein Interactions. *Nat. Commun*. **2020**, 11(1), 3954.
2. De Vries-van Leeuwen, I.J.; da Costa Pereira, D.; Flach, K.D.; Piersma, S.R.; Haase, C.; Bier, D.; Yalcin, Z.; Michalides, R.; Feenstra, K.A.; Jiménez, C.R.; de Greef, T.F.; Brunsveld, L.; Ottmann, C.; Zwart, W.; de Boer, A.H. Interaction of 14-3-3 proteins with the estrogen receptor alpha F domain provides a drug target interface. *PNAS* **2013**, 110 (22), 8894-8899.
3. Sijbesma, E.; Hallenbeck, K.K.; Leysen, S.; de Vink, P.J.; Skóra, L.; Jahnke, W.; Brunsveld, L.; Arkin, M.R.; Ottmann, C. Site-Directed Fragment-Based Screening for the Discovery of Protein-Protein Interaction Stabilizers. *J Am Chem Soc.* **2019,** 141 (8), 3524-3531.
4. Ballone, A.; Centorrino, F.; Wolter, M.; Ottmann, C. Structural characterization of 14-3-3ζ in complex with the human Son of sevenless homolog 1 (SOS1). *J Struct Biol*. **2018**, 202 (3), 210-215.
5. Kondo, Y.; Ognjenović, J.; Banerjee, S.; Karandur, D.; Merk, A.; Kulhanek, K.; Wong, K.; Roose, J.P.; Subramaniam, S.; Kuriyan, J. Cryo-EM structure of a dimeric B-Raf:14-3-3 complex reveals asymmetry in the active sites of B-Raf kinases. *Science*. **2019**, 366 (6461), 109-115.
6. Sijbesma, E.; Somsen, B.A.; Miley, G.P, Leijten-van de Gevel, I.A.; Brunsveld, L.; Arkin, MR.; Ottmann, C. Fluorescence Anisotropy-Based Tethering for Discovery of Protein-Protein Interaction Stabilizers. *ACS Chem Biol.* **2020**,15 (12), 3143-3148.
7. Centorrino, F.; Ballone, A.; Wolter, M.; Ottmann, C. Biophysical and structural insight into the USP8/14-3-3 interaction. *FEBS Lett.* **2018**, 592 (7),1211-1220.
8. Cossar, P.J.; Wolter, M.; van Dijck, L.; Valenti, D.; Levy, L.M.; Ottmann, C.; Brunsveld, L. Reversible Covalent Imine-Tethering for Selective Stabilization of 14-3-3 Hub Protein Interactions. *J Am Chem Soc.* **2021**, 143 (22), 8454-8464.
9. Wolter, M.; Valenti, D.; Cossar, P.J.; Levy, L.M.; Hristeva, S.; Genski, T.; Hoffmann, T.; Brunsveld, L.; Tzalis, D.; Ottmann, C. Fragment-Based Stabilizers of Protein-Protein Interactions through Imine-Based Tethering. *Angew Chem Int Ed Engl*. **2020**, 59 (48), 21520-21524.
10. Geertjens, N. H. J.; de Vink, P. J.; Wezeman, T.; Markvoort, A. J.; Brunsveld, L. A General Framework for Straightforward Model Construction of Multi-Component Thermodynamic Equilibrium Systems. *bioRxiv* **2021**, 2021.11.18.469126.
11. Ballone, A.; Lau, R.A.; Zweipfenning, F.P.A.; Ottman, C. A new soaking procedure for X-ray crystallographic structural determination of protein–peptide complexes. *Acta Cryst* **2020**, F76, 501.507
12. Potterton, L. et al. CCP4i2: the new graphical user interface to the CCP4 program suite. *Acta Crystallogr. Sect. D Struct. Biol*. **2018**, 74, 68–84.
13. Clabbers, M. T. B. et al. Electron diffraction data processing with DIALS. *Acta Crystallogr. Sect. D Struct. Biol.* **2018**, 74, 506–518.
14. Evans, P.R.; Murshudov, G.N. How good are my data and what is the resolution? *Acta Crystallogr. D. Biol. Crystallogr.* **2013**, 69, 1204-14.
15. Evans, P.R. An introduction to data reduction: space-group determination, scaling and intensity statistics. *Acta Crystallogr. D. Biol. Crystallogr.* **2011**, 67, 282-292.
16. Vagin, A.; Teplyakov, A. Molecular replacement with MOLREP. *Acta Crystallogr. D. Biol. Crystallogr.* **2010**, 66, 22-25.
17. Long, F. et al. AceDRG : a stereochemical description generator for ligands. *Acta Crystallogr. Sect. D Struct. Biol.* **2017** 73, 112–122.
18. Emsley, P.; Lohkamp, B.; Scott, W.G. & Cowtan, K. Features and development of Coot. Acta Crystallogr. Sect. D Biol. Crystallogr. **2010**, 66, 486–50.
19. Murshudov, G.N. et al. REFMAC5 for the refinement of macromolecular crystal structures. Acta Crystallogr. D. Biol. Crystallogr. **2011**, 67, 355–67.
20. Kovalevskiy, O. et al. Overview of refinement procedures within REFMAC5: utilizing data from different source. *Acta Crystallogr. Sect. D Biol. Crystallogr.* 74, 215-227 (2018).
21. Afonine, P.V. et al. Towards automated crystallographic structure refinement with phenix.refine. *Acta Crystallogr. Sect. D Biol. Crystallogr.* 68, 352–367 (2012).
22. Adams, P.D. et al. PHENIX: a comprehensive Python-based system for macromolecular structure solution. *Acta Crystallogr. D. Biol. Crystallogr.* 66, 213-221 (2010).
23. Schrodinger LLC. The PyMOL Molecular Graphics System, Version 2.2.3. (Schrödinger LLC, 2015)
